# Identifying potential survival strategies of HIV-1 through virus-host protein interaction networks

**DOI:** 10.1186/1752-0509-4-96

**Published:** 2010-07-15

**Authors:** David van Dijk, Gokhan Ertaylan, Charles AB Boucher, Peter MA Sloot

**Affiliations:** 1 Computational Science, University of Amsterdam, Sciencepark 107, 1098 XG Amsterdam, The Netherlands; 2 Department of Virology, Erasmus Medical Center, 's-Gravendijkwal 230, 3015 CE Rotterdam, The Netherlands

## Abstract

**Background:**

The National Institute of Allergy and Infectious Diseases has launched the HIV-1 Human Protein Interaction Database in an effort to catalogue all published interactions between HIV-1 and human proteins. In order to systematically investigate these interactions functionally and dynamically, we have constructed an HIV-1 human protein interaction network. This network was analyzed for important proteins and processes that are *specific *for the HIV life-cycle. In order to expose viral strategies, network motif analysis was carried out showing reoccurring patterns in virus-host dynamics.

**Results:**

Our analyses show that human proteins interacting with HIV form a densely connected and central sub-network within the total human protein interaction network. The evaluation of this sub-network for connectivity and centrality resulted in a set of proteins essential for the HIV life-cycle. Remarkably, we were able to associate proteins involved in RNA polymerase II transcription with hubs and proteasome formation with bottlenecks. Inferred network motifs show significant over-representation of positive and negative feedback patterns between virus and host. Strikingly, such patterns have never been reported in combined virus-host systems.

**Conclusions:**

HIV infection results in a reprioritization of cellular processes reflected by an increase in the relative importance of transcriptional machinery and proteasome formation. We conclude that during the evolution of HIV, some patterns of interaction have been selected for resulting in a system where virus proteins preferably interact with central human proteins for direct control and with proteasomal proteins for indirect control over the cellular processes. Finally, the patterns described by network motifs illustrate how virus and host interact with one another.

## Background

Recent advances in high throughput genome-wide screening techniques have increased not only the amount of generated data, but also its quality. In combination with the completion of the human genome project, this has led to early expectations of revolutionizing medicine. However, as often is the case in life science, the devil is in the details. We have learned that before we can efficiently use genome-wide data for developing the next generation of drugs and treatments we have to revolutionize the way we use our data [1]. Since we have recognized that we are not yet equipped with the right tools to interpret this unprecedented amount of data we have been building large databases where data is waiting to be processed into information. Today interpreting this data stands as the grand challenge for bioinformatics in the post-genomic era.

Meanwhile, hoping to solve this problem, we have been broadening our view and have been looking elsewhere for answers. One of these is the field of network science. This relatively new field has emerged from graph theory and physics and has proved to be a powerful method for the mathematical representation, visualization and analysis of complex data that involves many interacting components. In this area powerful concepts have been developed, such as network centrality, scalability and network motifs, that have enabled us to understand a system through its network topology [2-9]. Subsequently many fields have benefited from these advances. For example in epidemiology the mapping of human interactions into social networks gave insight into how sexually transmitted diseases spread in a population [10-12]. In developmental biology the representation of interactions among different genes as gene regulatory networks has been widely accepted [13-17] and in social sciences the analysis of human mobility patterns using a human interaction network helped us shed light on the dynamics of our society [18].

However, the field of virology has not yet received the full attention it deserves from network research, despite the availability of data and ready to use scientific methodology. Only recently Dyer and colleagues have described a network between human proteins interacting with viruses and other pathogens based on manually curated data from literature as well as publicly available databases [19]. In their work they give an overview of the common interacting proteins of viruses such as HIV, Incense and Measles to pathogen groups like Toxoplasma and Plasmodium. Their findings emphasize that pathogens preferentially interact with two kinds of proteins: **hubs **(ones that interact with many other proteins) and **bottlenecks **(ones that lie on many shortest paths). They also provide evidence from Gene Ontology (GO) annotation that different sets of pathogens target the same processes even though they interact with different proteins. One remarkable feature of their data is that it is highly biased towards HIV interactions. Approximately eighty percent of all interactions are specific to Human Immunodeficiency Virus (HIV).

### Human Immunodeficiency Virus

Human immunodeficiency virus (HIV) is recognized to be responsible for one of the most destructive pandemics in recorded history. It causes thousands of deaths and substantially decreases the life quality of millions of individuals each year, most of which live in Sub-Saharan Africa.

Since the first isolation of HIV in 1981, scientists are investigating every aspect of the virus hoping to find a vaccine. Genomic research has revealed that HIV has a compact genome, which consists of nine open reading frames (leading to nine primary translation products) that code for fifteen different translational products, represented by nineteen proteins. Most of the coding regions of HIV overlap, except for the genes *rev *and *tat *that are split by introns.

Despite the compactness of its genome, HIV has a very high nucleotide substitution rate, several million times faster than one of the average eukaryotic genome. Such a high substitution rate enables a virus population to exist in a cloud of genotypes called *quasispecies *and to rapidly adapt to environmental changes by means of this diversity. Varying conditions such as different humoral and innate immune system responses within and between hosts or varying treatment regiments result in selection pressures therefore shifting the dominant virus genotype [20]. This led to the understanding that *the persistence of the virus in host relies on the complex web of interactions it has, rather than the fitness of its structural components*. In other words, HIV's strategy for dealing with environmental stress lies in its ability to change its structural components while maintaining their function. This is also the main reason why it is unlikely that a universal vaccine will be developed using conventional methods like targeting anchor proteins. Therefore, before we can expect to start developing a cure, we need to invest more in the understanding of the interplay between the virus and the host.

Describing an interplay between two systems requires the choice of an appropriate level at which the interactions will be studied. Since many HIV-human interactions have been studied on proteins, the protein interaction level appears to be the most suitable candidate. Recently many of these interactions have been collected in the HIV-1, Human Protein Interaction Database of the National Institute of Allergy and Infectious Diseases [21]. In this database HIV proteins, interacting human proteins as well as their interaction type are collected and organized (See Table 1). A general statistical analysis of this database has been performed recently [21,22].

**Table 1:** Fourteen most frequent types of interactions between HIV and human proteins.

*interaction*	*frequency*	*interaction*	*frequency*
interacts with	575	processed by	99
upregulates	486	regulated by	99
Binds	411	phosphorylated by	65
Activates	365	enhances	62
Inhibits	270	cleaves	61
downregulates	262	induces phosphorylation of	53
inhibited by	188	stimulates	53

In addition to the NCBI database there are three other independent data-sets available as a result of small interfering RNA (siRNA) screens [23-25]. However, there is surprisingly little overlap between these four resources.

A very recent review by Bushman et al. addresses this issue by comparing the results of these three siRNA screens [26]. There were 34 genes called in at least two siRNA screens where as little as three genes were common in all three screens. Furthermore, of the 34 genes on two or three lists, only 11 were reported in the NCBI database. They have explained several reasons that could contribute to this variation. In addition they have included the interactions from NCBI database and other related work to assemble a "*host-pathogen*" interaction network. The analysis of this all-combined host-pathogen network revealed ten clusters that are identified with a distinct biochemical or cellular function. The clusters that were identified not only confirm understanding of some known processes such as immune response and *tat *activation/transcriptional elongation but also suggest the existence of new processes previously overlooked such as proteasome and mediator complex activity.

Nevertheless there are two important shortcomings associated with siRNA screening. First, the siRNA method can not be used to identify genes if their knockdown is toxic (i.e. resulting in cell death). Hence the method can be argued to be biased towards the Identification of genes that have a phenotype, yet on the periphery of a pathway within the total HIV-1 Human interactome Second, it does not explain the type of interaction that the suggested gene might have with HIV proteins. Therefore we argue that if one aims to identify "*core proteins*" involved in important processes for viral survival and also wants to analyze resulting dynamics, one has to rely on relatively less-biased and well annotated data such as the NCBI database. However the quality of the published manuscripts differ among those present in the database. In this report, all individual calls reporting interactions are treated equally for computational analyses.

### HIV-1, Human Protein Protein Interaction Network and Analysis

In the remaining of this paper we introduce the HIV-1 Human Protein-Protein Interaction Network based on the database by the National Institute of Allergy and Infectious Diseases (NIAID) called HIV-1, Human Protein Interaction Database. In the results section we present our findings from network centrality and network motif analysis. In the discussion section we discuss the analysis of network topology and patterns that has led to the Identification of HIV specific proteins and processes associated with viral survival. In the methods section we explain how our network was inferred and annotated with publicly available human protein interaction data and gene ontology (GO) terms. Subsequently, newly developed algorithms are described in the methods section.

## Results

### Connectivity Analysis

The National Institute of Allergy and Infectious Diseases' (NIAID) HIV-1, Human Protein Interaction Database offers comprehensive data on nineteen HIV proteins (fifteen structural and four intermediate proteins) interacting with 1452 human proteins via 3959 interactions. The most frequent types of these interactions are summarized in Table 1 with their frequency. We can see that regulatory (up-regulates, down-regulates, regulated by) and activation/inhibition (activates, inhibits, inhibited by) are among the most common interactions.

**Figure 1:**
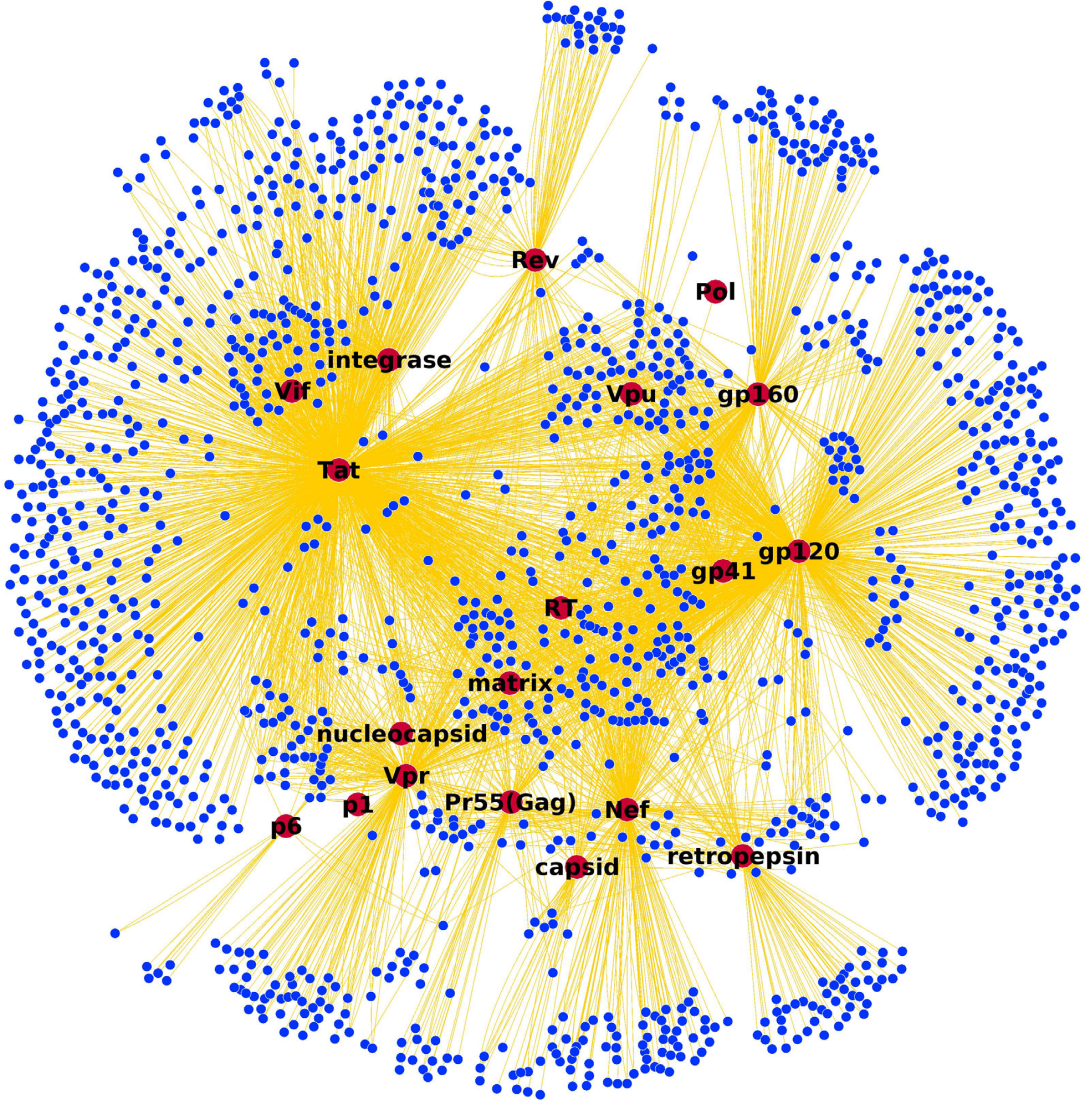
**HIV-Human protein interaction network**. Nineteen HIV proteins that interact with 1452 human proteins through 3959 interactions. Blue nodes are human proteins and red nodes are HIV proteins. Visualization is done with the Cytoscape [54] software using the spring layout algorithm.

We have inferred an HIV-1 Human Protein interaction network from this data (see additional files 1, 2, 3 and 4). A visualization of the network can be seen in Figure 1. It is apparent from this figure that some HIV proteins have many more interactions than others and some of the human proteins are interacting with more than one HIV protein. Furthermore we can state that these interactions are also different in nature. In order to distinguish between two different functional levels of interaction we have divided the total network into two distinct directed sub-networks by placing all regulatory interactions (upregulation/downregulation) in one sub-network (HIV-host regulatory network) and activation-inhibition related interactions in another (HIV-host activation/inhibition network). In order to study the influence of the pathogen on the host and vice versa only directed interactions were considered - non-directed interactions, like "binds" and "interacts with" were left out. The annotations in the database are somewhat ambiguous, i.e. the regulatory interactions not only point to transcriptional regulatory processes and activation/inhibition interactions not exclusively are signaling interactions. Therefore, the concepts that we use for both subnetworks (regulation and activation/inhibition) have a broader meaning and should not be directly interpreted as transcriptional regulation and signaling networks. Nonetheless, semantically a distinction between the two can be made. Also, regulation and activation/inhibition between proteins usually act at different time-scales and on different molecular levels, even though they are not decoupled processes but are co-occurring in many pathways in the cell. For this reason distinguishing between these two functional sub-networks also gives us the opportunity to study the different levels of involvement of the HIV proteins in these sub-networks. We have therefore conducted a connectivity analysis for each HIV protein in both networks to address this issue.

**Figure 2:**
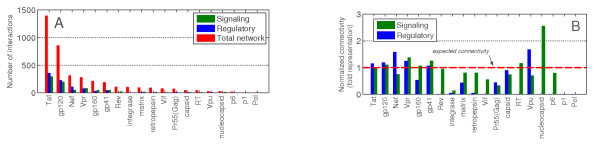
**A: Number of HIV-human interactions for each HIV protein, B: Normalized relative number of HIV-human interactions for each HIV protein**. The y-axis enumerates the n-fold representation of interactions per HIV protein divided by the relative number of interactions of the respective protein in the total network. An n-fold representation of *n *> 1 shows an over-representation, whereas *n *< 1 signifies an under-representation.

Figure 2 shows a bar-plot of all nineteen HIV proteins and their connectivity in the total, regulatory and activation/inhibition network (see additional file 5 for a connectivity distribution including "binds" interactions). From this we observe a non-uniform distribution of human interactions with HIV proteins, suggesting distinct functionalities (see Figure 2-A). It is not surprising that Tat has many connections given its central role as transactivator in promoting viral transcription and its effect on disease progression by interacting with neighboring cells after being released to the intercellular medium [27]. Gp120 also has many interactions due to its essential function in facilitating cell entry in different cell types [28] and Gp120 shedding of the virus [29]. Gp120 shows a similar distribution as Tat and is found in infected cells as well as in the intercellular space. The structural proteins P1, P6 and Nucleocapsid as well as unspliced Pol only have a small number of interactions. This is most likely due to their specific involvement in cellular processes and their presence which is confined to the intracellular space [30]. Proteins like Tat and Gp120 have been studied extensively, possibly because of their central role in HIV infection and their potential as drug targets.

This explains their overrepresentation in Figure 2-A. To correct for this bias we have calculated a relative connectivity distribution of the activation/inhibition and regulatory sub-networks using normalization (see section methods for details). This allows for direct comparison of connectivities between HIV proteins and between the two sub-networks (see Figure 2-B).

One interesting aspect to note is that the HIV proteins that are exposed to the host environment (in the case of Gp120 and Gp41 by expression on the virus's outer membrane or secretion to the extracellular environment in the case of Tat and Vpr) have almost exactly the same number of interactions expected from their overall connectivity. In other words, they show no sign of specification for the activation/inhibition or regulatory networks. The unspliced Gp160 on the other hand, is under-represented in the regulatory network. Furthermore, HIV Integrase has very little involvement in activation/inhibition and virtually no involvement in the regulatory processes. No significant correlation was observed with the amino-acid length of each viral protein and its involvement in any of the networks (Data not shown). We hypothesize that HIV-1 interacting central human proteins may play a significantly more important role than non-central ones in the life cycle of HIV-1. Therefore, we have conducted a similar connectivity analysis for the human proteins from the total network. From the point of view of the human proteins we once again observe a non-uniform distribution of interactions. (Data not shown) Table 2 shows the ten most connected HIV Dependency Factors (HDFs) with varying degrees. Not surprisingly three kinases Atmpk1, Prkca and Mapk3 (which take part in a wide variety of cellular processes) and the immune system cytokine Ifng are identified as the most connected proteins.

**Table 2:** Top ten highest connected HDFs, considering only HIV-HDF connections.

*Name*	*Definition*	*Degree*
ATMPK1 [GenBank:NP_000537.3]	mitogen-activated protein kinase 1	10
IFNG [GenBank:NP_009225.1]	interferon, gamma	9
PRKCA [GenBank:NP_000312.2]	protein kinase C, alpha	9
MAPK3 [GenBank:NP_068810.2]	mitogen-activated protein kinase 3 isoform 1	9
ACTB [GenBank:NP_852664.1]	beta actin	8
ACTG1 [GenBank:NP_002458.2]	actin, gamma 1 propeptide	8
HLA-A [GenBank:NP_004371.2]	major histocompatibility complex, class I, A precursor	8
CD4 [GenBank:NP_002077.1]	CD4 antigen precursor	8
IL10 [GenBank:NP_001420.2]	interleukin 10 precursor	7
IFNA1 [GenBank:NP_002219.1]	interferon, alpha 1	7

**Figure 3:**
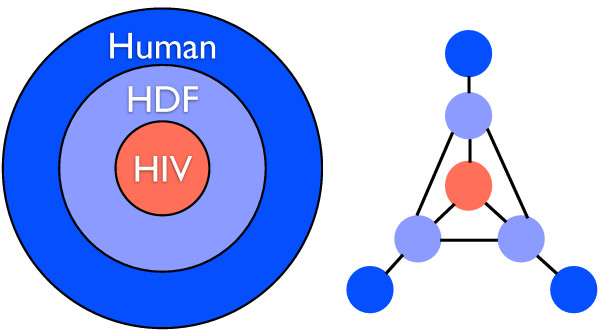
**HIV proteins interact with HIV dependency factors (HDFs) which in turn interact with human non-HDF proteins**. Understanding HIV-host interaction requires the understanding of the HDF network and its position within the total human protein interaction network.

**Figure 4:**
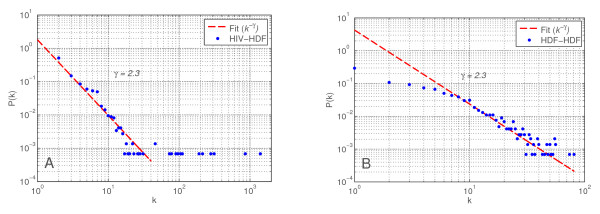
**Degree distributions of HDFs on a log-log scale**. *P*(*k*) is the fraction of nodes with degree k. A: Only connections of HDFs to HIV proteins. B: Only HDF-HDF connections. Both distributions were fitted with a power law (*P*(*k*) = *k*^-γ^) with A: *γ *= 2.3, and B: *γ *= 2.3, showing the scale-free nature of both networks.

HIV has many interactions with human proteins, and on many levels. Yet these interactions become meaningful only when we can put them into context. Therefore we have enriched our HIV-1 human protein interaction network with interactions from human protein interaction databases BIND, BioGRID and HPRD (see methods). First we have included interactions between the HDFs (the local network) and interactions with non-HDF human proteins (the global network). The resulting network is a human protein interaction network where HIV interacting human proteins or HDFs are connected to each other and also to non-HDF human proteins. Figure 3 shows an abstract representation of the structure of this network. In Figure 4 two degree distributions of the networks are shown. In Figure 4-A, we can see the degree distribution of HDFs considering only interactions with HIV proteins. In Figure 4-B, we only consider the HDF-HDF interactions. On both graphs the power-law distribution indicates the scale-free nature of the networks, caused by a topology where most proteins have few connections, but a small number of proteins are highly connected, thus acting as hubs. Networks with scale-free properties are thought to be resilient to random perturbations and are therefore robust [5].

#### Metrics: Centrality

We hypothesize that central genes or proteins in the human protein interaction network are more likely to be important players in the life cycle of the virus than non-central ones. Therefore, after constructing the HIV-1 human protein interaction network we have measured three types of network centrality: degree, betweenness and eigenvector centrality on both local and global networks.

#### HDF sub-network is Central

To determine the importance of individual HDFs regarding connectivity in the total human protein interaction network we define two scores: a hub score and a bottleneck score. The degree and the eigenvector centrality of a protein describe how well it is connected to other proteins (see methods for a detailed description of both measures). For this reason we have associated the term "hub" with these measures. Network centrality encompasses several different notions in connectivity analysis, degree and eigenvector centrality being two of them. Another concept that is used to describe the position in a network is by looking at paths rather than connections. Betweenness centrality is used to measure the centrality of a node in the network by counting the number of shortest paths that go through that node. In other words, how many shortest paths would increase in length if the node is removed from the network [31]. See the methods section for a definition of and earlier work on network ranking [19,32]. Table 3 shows these centrality metrics measured for the total human protein interaction network (global) and the HDF sub-network (local). Comparison of the HDF network with the total human protein interaction network using a Kolmogorov-Smirnov test shows that the measured degrees, eigenvector centralities and betweenness scores in the local and global network are not from the same distribution (see $P_{value}^{ks}$ in Table 3). Because the Kolmogorov-Smirnov test was performed one-sided, we can conclude that the local network is significantly more central than the global network with respect to the three metrics. This indicates a densely connected HDF network that takes on a central position in the whole human protein interaction network. Subsequently, this shows that the human proteins interacting with HIV tend to be involved in other important processes as well.

**Table 3:** Mean values of centrality measures on HDFs and on proteins of the whole human protein interaction network, with standard deviations between brackets.

*μ (σ)*	*HDF network*	*total human network*	$P_{value}^{ks}$**(*HDF > total*)**
degree	16 (25)	6 (11)	2.42·10^-97^
betweenness	63·10^3 ^(19·10^4^)	17·10^3 ^(79·10^3^)	9.22·10^-63^
eigenv. centr.	0.049 (0.10)	0.013 (0.04)	3.08·10^-87^

$P_{value}^{ks}$ is calculated from a one-sided Kolmogorov-Smirnov test with alternative hypothesis: HDF network > total human network regarding degree, betweenness and eigenvector centrality. In the HDF network each node has approximately three times more connections (16 in HDF vs 6 in the total human network), four times higher betweenness (63·10^3 ^in HDF vs 17·10^3 ^in human) and a four times higher eigenvector centrality score (0,049 in HDF vs 0,013 in human). The significant higher centrality of the HDF sub-network shows that it takes on a central position within the total human protein interaction network.

#### Hubs

We define a *hub *as a protein with high degree and eigenvector centrality (see methods section). Table 4 shows the proteins that were commonly identified as central nodes by both of these metrics.

**Table 4:** Set of proteins that are found to be hubs by both the degree and eigenvector centrality metrics.

*Name*	*Definition*	*Degree*	*Eigenv. centr.*
TP53 [GenBank:NP_000537.3]	tumor protein p53	93	1.00
BRCA1 [GenBank:NP_009225.1]	breast cancer 1, early onset isoform 1	74	0.98
ESR1 [GenBank:NP_000116.2]	estrogen receptor 1	59	0.83
CREB1 [GenBank:NP_004371.2]	CREB binding protein isoform a	58	0.81
RB1 [GenBank:NP_000312.2]	retinoblastoma 1	58	0.72
RELA [GenBank:NP_068810.2]	v-rel reticuloendotheliosis viral oncogene homolog A	57	0.75
SRC [GenBank:NP_005408.1]	proto-oncogene tyrosine-protein kinase SRC	57	0.53
TBP [GenBank:NP_005635.1]	TATA box binding protein	56	0.61
MYC [GenBank:NP_002458.2]	myc proto-oncogene protein	55	0.67
JUN [GenBank:NP_002219.1]	jun oncogene	51	0.72
EP300 [GenBank:NP_001420.2]	E1A binding protein p300	51	0.69

The top one percent of highest ranked proteins are shown here.

Table 4 summarizes the top one percent of the highest ranked HDFs in the total network. We notice from this table that both centrality metrics result in very similar sets of top ranked proteins. The extended table with the top five percent of proteins identified with different measures can be found in the additional file 6. We can see that P53, Brca-1 and Retinoblastoma-1 have been identified as being highly central by both metrics. This result is not surprising since all three are well established oncogenes and have been extensively studied. Therefore their connections with other proteins are expected to be better documented.

#### Bottlenecks

We define a protein with high betweenness score as a *bottleneck *[19].

Table 5 shows the top one percent of proteins that are called bottlenecks. Once again, highly documented proteins such as tumor protein Tp53, Ubiquitin C (UBC), Grb2 and Brca-1 are identified as the highest ranked proteins.

**Table 5:** Top one percent of proteins that have the highest score from the betweenness centrality metric.

*Name*	*Definition*	*Betweenness Score*
TP53 [GenBank:NP_000537.3]	tumor protein p53	44050
UBC [GenBank:NP_066289.2]	ubiquitin C	36458
GRB2 [GenBank:NP_002077.1]	growth factor receptor-bound protein 2 isoform 1	22792
BRCA1 [GenBank:NP_009225.1]	breast cancer 1, early onset isoform 1	21622
SRC [GenBank:NP_005408.1]	proto-oncogene tyrosine-protein kinase	21568
EGFR [GenBank:NP_005219.2 ]	epidermal growth factor receptor isoform a	20472
STAT3 [GenBank:NP_644805.1]	signal transducer and activator of transcription 3 isoform 1	18503
ESR1 [GenBank:NP_000116.2]	estrogen receptor 1	18424
RB1 [GenBank:NP_000312.2]	retinoblastoma 1	16777
PIK3R1 [GenBank:NP_852664.1]	phosphoinositide-3-kinase, regulatory subunit, polypeptide 1 isoform 1	16048
POLR2A [GenBank:NP_000928.1]	DNA directed RNA polymerase II polypeptide A	15896
MYC [GenBank:NP_002458.2]	myc proto-oncogene protein	15608
SP1 [GenBank:NP_612482.2]	Sp1 transcription factor	14620
RELA [GenBank:NP_068810.2]	v-rel reticuloendotheliosis viral oncogene homolog A	14114
SHC1 [GenBank:NP_003020.2]	Src homology 2 domain containing transforming protein 1 isoform p52Shc	14011

#### Identification of host factors that are specific to HIV infection

It is not surprising that from our centrality analysis the proteins that are important for the functioning of a cell are also crucial for the viral survival. The question that remains is "Are there HIV specific processes that are crucial for viral existence but not as important for the cell?"

**Figure 5:**
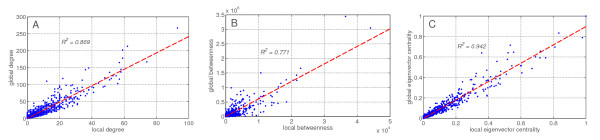
**A: Local degree versus global degree, with an *R*^2 ^of 0.869**. B: Local versus global betweenness, with an *R*^2 ^of 0.771. C: Local versus global eigenvector centrality, with an *R*^2 ^of 0.942.

In order to understand the relation between local (related to other HDFs) and global (related to all human proteins) properties of HDFs, we check whether high centrality in the HDF network is a predictor for high centrality in the total human protein interaction network. We plot the local against the global measures of all our metrics. In Figure 5 these three plots are shown, clearly signifying strong correlations.

Because of this strong correlation between local and global properties almost any protein that is identified as highly essential using a ranking based on local properties is also important globally. To counteract this effect we filter out proteins of global importance by re-ranking them using an adjusted metric (see methods for details).

In Table 6 the top one percent of proteins that are identified by both corrected degree and corrected eigenvector centrality metrics are shown. We observe from this table that highly studied oncogene products are replaced by the transcription machinery related proteins TBP-associated factor 1 isoform 1 (Taf1), Activating transcription factor 2 (Taf2), General transcription factor IIB (Gtf2b). This finding is important because it indicates that transcription is a vital process for HIV to synthesize proteins necessary for forming progeny.

**Table 6:** Set of proteins that are identified as central using both adjusted centrality metrics (degree and eigenvector centrality).

*Name*	*Definition*
TAF1 [GenBank:NP004597.2]	TBP-associated factor 1 isoform 1
ATF2 [GenBank:NP001871.2]	activating transcription factor 2
GTF2B [GenBank:NP001505.1]	general transcription factor IIB
CCND1 [GenBank:NP444284.1]	cyclin D1
STAT1 [GenBank:NP009330.1]	signal transducer and activator of transcription 1 isoform alpha
TBP [GenBank:NP003185.1]	TATA box binding protein
CDKN1A [GenBank:NP000380.1]	cyclin-dependent kinase inhibitor 1A
CEBPB [GenBank:NP005185.2]	CCAAT/enhancer binding protein beta

The top one percent of highest ranked proteins are shown here.

Table 7 is the result of normalizing the local betweenness measure by the global value. Proteasome subunits have gained significant importance and they constitute the three highest ranked proteins in terms of betweenness. Interestingly, in a recent review on the meta-analysis of genome-wide studies [26], proteasome has been reported to play an important role in HIV functioning. However, there is conflicting evidence regarding its action. Proteasome is predominantly reported in degradation of viral products in earlier literature [33,34] whereas recent genome-wide siRNA studies indicate a role in the facilitation of HIV infection [24-26]. Our result confirms the importance of proteasome and identifies it as a bottleneck. Since it is important to understand why proteasome appears as a crucial process specifically for HIV we have isolated all proteasomal proteins from the total network and included their first and second level interacting neighbors. The resulting network consists of three distinct clusters, where the first cluster clearly only involves proteasomal proteins. For functional annotation of the other clusters we have used the *DAVID bioinformatics resources *online service [35] and performed clustering with GO Biological Process (BP). After annotation, the second cluster is associated with regulation of metabolic process (80 percent of all proteins), and regulation of progression through cell cycle (49 percent of all proteins). The members of this cluster are proteins from highly connected oncogenes Tp53, Tp73, Brca-1 and Rb1. The third cluster is associated with signal transduction and cell communication (both 78 percent). These findings suggest that *the proteasomal proteins are identified as bottlenecks because they are connected to important cellular processes *mentioned above, as well as to the rest of the network. The network visualization of the proteasomal proteins with their first and second neighbors, the lists of proteins associated with each cluster and heat maps of associated GO terms can be found in the additional files 7, 8, 9, 10 and 11.

**Table 7:** Top ten bottlenecks after normalization.

*Name*	*Definition*	*Bottleneck Score*
PSMD6 [GenBank:NP055629.1]	proteasome (prosome, macropain) 26S subunit, non-ATPase, 6	0.44
PSMA2 [GenBank:NP002778.1]	proteasome alpha 2 subunit	0.25
PSMD10 [GenBank:NP002805.1]	proteasome 26S non-ATPase subunit 10 isoform 1	0.15
DHX9 [GenBank:NP001348.2]	DEAH (Asp-Glu-Ala-His) box polypeptide 9	0.08
CD4 [GenBank:NP000607.1]	CD4 antigen precursor	0.07
CD82 [GenBank: NP002222.1]	CD82 antigen isoform 1	0.07
IKBKE [GenBank:NP054721.1]	IKK-related kinase epsilon	0.06
PTPRC [GenBank:NP002829.2]	protein tyrosine phosphatase, receptor type, C isoform 1 precursor	0.06
A2M [GenBank:NP000005.2]	alpha-2-macroglobulin precursor	0.06
CCR5 [GenBank:NP000570.1]	chemokine (C-C motif) receptor 5	0.05

One remark is that "some of the virus-host interaction studies have been done on individual subunits of a complex, but at other times a complex is implicated in a virus-host interaction and all subunits of that complex are linked to a virus protein even though only a few subunits might be involved in the interaction. This might lead to spurious over-represented motifs." On the other hand, if those data describing interaction of complexes rather than individual subunits is discarded this might lead to an under-representation of complexes which would in reality be present in the motif analysis. We have chosen to include these in favor of over-representation of motifs since the HIV-1 human protein interaction data is already sparse.

### Network Motifs

Complex networks in general and biological networks specifically have been found to consist of small recurring patterns, so-called network motifs [2,4,36]. These building blocks have been used to study the structure and dynamic behavior of networks.

**Figure 6:**
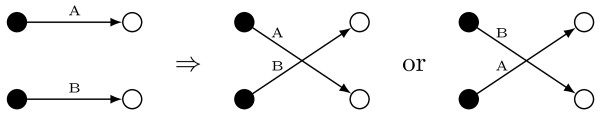
**Diagram representing the rewiring method used by the randomization algorithm**. Two random edges are chosen and either the sources or the targets are switched with equal probability.

**Figure 7:**
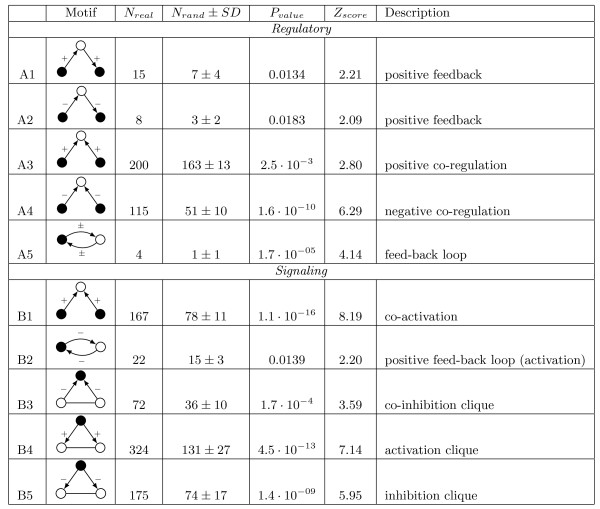
**Significantly over-represented network motifs in HIV-host protein interaction network**. Black nodes are HIV proteins and white nodes are human proteins. Interactions can either be activations/up-regulations (+), inhibitions/down-regulations (-), activation/inhibition/regulation (±), or both (arrow without sign). *N*_*real *_is the number of specific motifs found. *N*_*rand *_± *SD *is the average number and standard deviation of the motif found in one thousand randomized networks. *P*_*value *_is the probability that *N*_*real *_or more motifs are found in the randomized networks. *Z*_*score *_is the number of standard deviations *N*_*rand *_differs from *N*_*real*_. Network motifs were classified as significant when *P*_*value *_< 0.02 and *Z*_*score *_> 2.

The motif analysis was carried out on the regulatory and activation/inhibition subnetworks (inferred from the HIV-1 Human protein interaction network) by comparing the subnetworks with one thousand randomized networks, which were created by randomly rewiring the original networks (see Figure 6 and section Methods for details on the rewiring algorithm). This resulted in a number of significant motifs. In Figure 7 a selection (see additional file 12 for the complete table) of motifs are listed that were found to be significant (*Z*_*score *_> 2, *P*_*value *_< 0.02). Next we describe the types of motifs found.

#### Self-regulation, feedback

**Figure 8:**
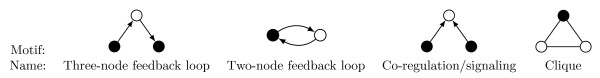
**General types of motifs found in the HIV-human protein interaction network**. Black nodes represent HIV proteins, white nodes represent human proteins.

A feedback pattern was found for both two and three node motifs, consisting of one human protein and one or two HIV proteins. The three node feedback loop motif (see Figure 8), identified as indirect self-regulation, is a pattern where an HIV protein regulates or signals a human protein that in turn regulates/signals another HIV protein. As the two HIV proteins are part of the same organic structure (the HIV pathogen) we observe a process of self-regulation or activation/inhibition (feedback) depending on the nature of the interactions. The two node feedback loop (self-regulation motif, see Figure 8) consists of one HIV protein that regulates/signals a human protein that in turn regulates/signals the HIV protein. The specific type of interactions between the proteins is what determines the nature of the feedback, e.g. two up-regulations result in a positive feedback, as well as two down-regulations. On the other hand a negative feedback will be the result of one up- and one down-regulation. The three node feedback pattern was observed in seven different regulatory motifs and in one activation/inhibition motif, additionally a two node feedback motif was found in the regulatory network as well as in the activation/inhibition network (see additional file 12).

#### Co-regulation

Co-regulation, or co-activation/inhibition is what we describe as two HIV proteins regulating/activation/inhibition one human protein (see Figure 8). The two interactions can be of the same type (e.g. both up-regulation, or inhibition), where they can show a potential redundancy in the system. Of the co-regulation motif we found six types of regulatory and two types of activation/inhibition motifs to be significantly over-represented.

#### Clique

Inclusion of interactions between HDFs (collected from human protein interaction databases, see methods section) gives the ability to study the relationship between HDFs that have a common interacting HIV protein. The network motif that is associated with this pattern is what we identify as a "clique" (see Figure 8). Traditionally the term clique has been used to denote a group of fully interconnected nodes [37], but has also been used to describe network motifs of the fully connected three node sub-graph [2]. In this work we study such a clique that consists of two human proteins and one HIV protein. As the interactions between HIV and human nodes have directionality a number of different clique patterns arise, similar to the ones without HDF-HDF interactions.

A feed-forward type [2-4,36] (or self-regulatory) motif occurs when two connected HDFs are also (indirectly) interacting via an HIV protein. Co-regulation (or activation/inhibition) is also observed in the clique. Two interacting human proteins both also regulate/signal the same HIV protein. Again when the two interactions are of the same type this might indicate a redundancy (see Discussion). Nine different clique patterns were observed in the regulatory network and five in the activation/inhibition network. We have also conducted a Gene Ontology analysis for each motif that was identified (see additional file 12).

## Discussion

In this study we have analyzed a pathogen-host protein interaction network in an effort to relate network topology to biological functioning. Topologically central proteins have shown to be crucial for HIV functioning and network motifs appear to be the result of the complex virus-host interplay. In this section we discuss these results from the network centrality metrics and the network motif analysis.

### Network Centrality

#### HIV Human Protein Interaction Network Meta-Analysis

First we have conducted a meta-analysis of the HIV-human protein interaction network to examine the distribution of interactions among HIV proteins as well as HDFs. Network analysis identified key components in the life cycle of HIV.

The normalized relative connectivity analysis revealed involvement of viral proteins in distinct sub-functions (activation/inhibition and regulatory).

*Integrase *is a viral enzyme that enables the viral genome to be integrated into the DNA of the host cell. In addition to this it is present at the time of the initial infection of a cell in only small amounts [38]. One can speculate that any dual function of activation/inhibition or regulatory nature would end up in reduced efficiency and probably early detection by the human immune machinery before completing the job. This might be the reason why it is involved in neither the activation/inhibition network, nor the regulatory network.

HIV proteins which are exposed to the extracellular environment (Gp120, Gp41, Tat and Vpr) have approximately an equal number of interactions inferred from their global connectivity in the total network. This is probably due to the large variety of function related to these proteins. It is indeed true for Tat and Vpr and possibly for Gp120, that they are *hyperactive *in terms of their role in different processes. All three proteins are also directly exposed to the extracellular factors such as antibodies. Gp41 on the other hand, is originally buried in the viral envelope and is exposed only after Gp120 binds to a CD4 receptor. In addition, Gp41 has been associated with a specific role in viral membrane fusion. So it is puzzling that Gp41 is sharing this generic connectivity profile. On the other side of the spectrum, viral enzymes *RT*, *retropepsin *and *integrase *all show interaction profiles that are highly specific for activation and inhibition interactions. These enzymes are reaction specific and functional changes are likely to be too costly for the virus, therefore might be favorable to keep these proteins uni-functional.

Similar connectivity analysis for human proteins revealed Mitogen-activated protein kinase 1 (Mapk1), Interferon gamma (Ifng) and Protein kinase C alpha (Prkca) and Mitogen activated protein kinase 3 (Mapk3) as the most HIV connected nodes in HIV-human protein interaction network, having degrees 10, 9, 9 and 9 respectively. Mapk1 is identified as the integration point for multiple pathways and takes part in a wide variety of cellular processes [39]. Ifng is an important cytokine for innate and adaptive immunity. Prkca and Mapk3 are both known to be involved in various critical cellular processes. It is not unexpected that we find them to be over-represented in the HIV-1 human protein interaction network.

#### Centrality Analysis

Meta-analysis of the HIV-human protein interaction network revealed that HDFs interacting with HIV constitute a non-random sub-network (HDF network) in the human interactome. We employed three centrality measures (degree, betweenness and eigenvector centrality) to analyze the HDF sub-network in detail. We calculated the average centrality measures for the HDF network as well as the total human protein interaction network. It is clear that the HDF network is located topologically central in the human-protein interaction network and is significantly densely connected.

Hub analysis of the HDF network resulted in fifteen proteins that are found to be central for at least one of the two centrality metrics (degree and eigenvector centrality) where six of them were oncogenes.

Bottleneck analysis was conducted based on the betweenness centrality and resulted in a similar list to the hub analysis. Further inspection showed that both were also highly central in the total human protein interaction network.

We calculated the correlation between local and global centrality for each of the centrality metrics that resulted in high correlation for each measure. This means that the centrality assigned to each protein in the HDF network was a result of its high connectivity in the total network. To overcome this problem and identify HIV specific processes we have normalized each centrality measure from the HDF network by its global network counterpart. We observe from the normalized list that highly studied oncogenes are replaced by transcription factors, transcription factor sub-units (TBP) and transcription activators. This finding is important because although transcription is important for the cell, it is probably "the vital" processes for HIV to synthesize proteins necessary for forming progeny. It is important to note that in the normalized bottlenecks list, three proteasome subunits constitute the most important bottlenecks specific for the HDF network. Proteasome subunits were also identified as one of the important processes by Bushman et al. [26]. It is known that cellular proteasome can act negatively on HIV infection by destroying viral proteins but it is not clear what the overall effect is on the infection. Our results show that the importance of protease stems from the close interaction between vital proteins in regulation of gene expression and cell communication with proteasomal proteins. Therefore proteasome seems to connect the processes governed by these proteins and the rest of the HDF network. All biochemical reactions in the cell are dynamic and their equilibrium depends on the concentration of the substrates available. Proteasomes have a unique role in this scenario by being the regulator of the concentration of particular proteins. A strong line of evidence for HIV's exploitation of proteasomal pathways comes from the innate restriction host factors that inhibit viral replication at the cellular level. Human CD317/Tetherin and APOBEC proteins (APOBEC3G and APOBEC3F) have been identified to inhibit HIV replication and render resistance to HIV infection. There is growing evidence that HIV proteins Vpu and Vif accelerate proteasomal degradation of CD317/Tetherin [40-43] and APOBEC3G/F [44,45] respectively, thus suppressing their expression and overcoming the innate resistance. Strikingly, the human restriction factor tetherin mentioned above is not curated into the NIAID database. Yet, the importance of proteasomal degradation for HIV infection has been identified independently in this work. Given the critical role of HIV's Vif and Vpu in suppressing APOBEC3G/F and CD317 activity, we argue that pharmacologic compounds designed for restoring the activity of these intrinsic anti-viral factors in infected cells in-vivo, could have strong therapeutic benefits, and therefore deserve serious attention.

As a result, we hypothesize that *after infection, apart from degrading HIV proteins, re-prioritization of proteasomal pathways is an indirect control mechanism actively engaged by the virus to manage the concentrations of pivotal proteins in the cell*. We have shown that regulation of gene expression and cell communication are major processes that are directly linked to proteasome functioning.

### Network motifs

Traditionally networks of single systems have been studied using network motifs (e.g. gene regulatory network of yeast, see [3]). Discovered patterns, in terms of over-represented network motifs, hold information on network structure and dynamics of that system. HIV infection and it's life-cycle is based on the interplay between two systems, namely the virus itself and the human host. Consequently, network analysis using motifs results in understanding of dynamics and structure of interplay as opposed to the functioning of the two systems independently.

#### General patterns

By interpreting the inferred network motifs (see Figure 7 and 8) we achieve insight into this interplay. Self-regulation or feedback is a pattern that is commonly found in gene regulatory systems (see [2-4,36]). Generally these patterns indicate a response mechanism, where a signal such as a gene regulation (up-regulation, down-regulation) or a phosphorylation (activation, inhibition) of a protein A triggers a similar signal to protein B. In the two node case the source of the signal to A is B, thus potentially resulting in a positive or negative feedback loop. In the three node case (two different HIV proteins) interpretation is less trivial. When we consider all HIV proteins that make up the virus as a unity, we may consider the motif as a feedback or self-regulation. Since current available data is lacking information on interactions between HIV proteins, we are not able to interpret it as a loop. Yet interaction between HIV proteins, especially with the regulatory protein Tat, are known to be prevalent [27]. Therefore it is plausible to assume the existence of three node feedback loops.

One limitation of the network motif analysis is the absence of time (or causality) and spatial information associated with each event in the database. Therefore, reconstruction of pathway dynamics by means of network motifs is not possible. One way to overcome this problem, at least for some motifs, is to include interactions among human proteins that indicate shared compartments and time. For instance, co-regulation, specifically in the case of two of the same interactions, points to a potential redundancy. This only holds when we assume that the two similar interactions occur in a shared spatial and temporal frame, i.e. the interactions happen in the same cellular compartment and roughly at the same time. This assumption becomes more plausible when HDF-HDF interactions are incorporated, serving as proof for the co-occurrence in time and space, of the two proteins. Co-regulation that occurs within a clique thus more strongly points to redundancy. It is these redundancies that are known to contribute to the robustness of regulatory networks in general [46-48] and give evidence for a potential cause of the robust nature of HIV infections.

### Survival Strategy

Studying HIV-human interaction in terms of network motifs gives us the opportunity to reconstruct dynamics on the protein level. It is known that under selection pressure by the immune system the HIV virus undertakes a number of actions to evade this defense. This interplay where the host tries to undermine virus reproduction and where the virus evades immune response is the key concept for understanding virus-host relations.

Network motifs that have been found to be significantly over-represented, i.e. when their existence can not only be accounted for by randomness, show patterns that apparently have been selected for. By investigating these motifs individually we observe these strategies on the protein interaction level.

One of such motifs is a two node feedback loop, found in the HIV-host activation/inhibition network (see motif B2 in Figure 7). Significant over-representation of this network motif shows the inhibitory behavior of HIV proteins on human proteins that in turn inhibit the HIV protein. We therefore refer to these patterns as an "indirect positive feedback" and in this specific case "self-activation" as inhibition of an inhibitor results in (relative) activation. Closer inspection of all occurrences of this network motif shows that the HIV Tat and Gp120 protein and the human protein Interferon Gamma (Ifng) have the highest level of involvement. Gene Ontology analysis of the observed network motif indicates that the human proteins involved in the network motif are involved in immune response (see additional file 12).

Ifng, or type II interferon, is a cytokine critical for innate and adaptive immunity against viral and intracellular bacterial infections and for tumor control. The importance of Ifng in the immune system stems in part from its ability to inhibit viral replication directly, but most importantly derives from its immunostimulatory and immunomodulatory effects [49,50].

We want to acknowledge that the results presented in this paper are based on annotated protein interaction data from the NIAID database. This data varies strongly in quality and it can be argued to contain a bias as a result from translating individual reports into a structured database. Therefore the results presented above should be interpreted *qualitatively authentic *rather than quantitatively accurate. Nonetheless, the presented work is the first in the field, according to our knowledge, to incorporate network centrality analysis and network motifs in a virus-host protein interaction network. We encourage experimental testing of the results in this paper to study their potential role in HIV infection.

## Conclusions

We have demonstrated that infection with HIV results in re-prioritization of cellular processes such as transcription and proteasome activity. The primary success of the virus depends on the synthesis of new virions in a reasonable amount of time. This has to be accomplished before the infected cells are detected by patrolling CD8+ T cells or a humoral response has emerged. Therefore it is highly plausible that hijacking of the transcriptional machinery is one of the key processes that has a pronounced role post-infection.

In addition, proteasomes not only gain significant importance for the survival of the cell by degrading HIV proteins early in the infection, but arguably also for HIV, since they regulate the concentration of the innate antiviral host factors such as APOBEC3G/F and CD317 and can be targeted by HIV proteins Vpu and Vif.

We have shown that using network motifs one can identify recurring patterns that have consequences in the virus-host dynamics. Specifically, we observed patterns that show strategies of the virus used to evade the host immune system. Finally, we conclude that the survival of HIV within the host requires direct control of the cellular machinery via the pivotal human proteins and indirect control via the proteasomes. Network motifs and complex network theory provide a promising framework to study these dynamics.

## Methods

### NCBI Database to network

The NCBI HIV-Human Protein Interaction database is used to construct a protein interaction network. The obtained network consists of nineteen HIV proteins that interact with 1452 human proteins through 3959 interactions (See Figure 1.)In this protein interaction network nodes represent either HIV or human proteins and edges interactions between them. Because interactions between HIV and human proteins are annotated (see Figure 8) for most common interaction types), edges in our network are directed and have an interaction type. As interactions are only between HIV and human protein, the resulting network is bipartite.

### HIV protein connectivity

Figure 2 shows the connectivity of the nineteen HIV proteins in the HIV-Human protein interaction networks. Figure 2-A shows the absolute number of interactions per HIV proteins for each of the two subnetworks and the total network. Figure 2-B shows the normalized relative connectivity. This was achieved by first calculating the relative connectivity, by dividing the number of interactions for each protein and network by the total number of interactions in that network. Next the numbers were normalized by dividing the relative connectivity for each protein and each of the two subnetworks by the relative connectivity of that protein in the total network. This normalization permits the comparison of proteins and subnetworks.

### Human Protein interactions

To incorporate interactions between HDFs and between HDFs and human non-HDF proteins, data on protein interaction was collected from several databases (BIND, BioGRID, HPRD) and added to the network [51-53]. As a result the network consists of nineteen HIV proteins, 1,452 HDFs and 12,557 non-HDF human proteins, and 3,959 HIV-HDF interactions, 4,540 HDF-HDF interactions and 13,189 interactions between HDFs and non-HDF human proteins.

### Network visualization

Cytoscape [54] was used to visualize the HIV-1 human protein interaction network. The spring embedded layout algorithm was used for Figure 1.

### Network statistics

Network analysis and filtering was performed using IGraph [55], an R [56] package for complex network analysis.

The metrics that are used to rank HDFs according to their importance in the network are based on a number of network centrality measures (measured per node):

• Degree: number of connected edges, i.e. number of protein interactions

• Eigenvector centrality: measure of connectedness to other well connected nodes [57,58]

• Betweenness: number of shortest paths that go through the node [59,60]

In contrast to the degree, which is a measure of direct connectedness (number of interacting proteins in our case), the eigenvector centrality measures direct and indirect connectedness. Because well connected nodes contribute more to the score of their neighbors than low connected nodes, a protein with a relative high eigenvector centrality not just indicates high activity in terms of different interactions, but also points to activity in important pathways. The betweenness centrality, on the contrary, only measures pathway activity. A protein with high betweenness is positioned at a central location in the network, as relatively many shortest paths cross it. This does not necessarily imply well connectedness in terms of degree; a low connected protein might still have a high centrality. This way important "cross-roads" in the network can be identified, that would not have been noticed using standard degree analysis.

Using these three metrics we seek to measure the importance of human proteins that interact with HIV proteins (HDFs). In order to distinguish between HDFs that are important to whole human functioning and HDFs that are specifically important to the HIV life-cycle, we normalize our centrality ranking using a distinction between "local" and "global" metrics.

For instance, we define local degree of an HDF as the number of edges to other HDFs, and global degree of an HDF as the number of edges to any other human protein (including HDFs). So local degree measures connectivity within the HDF network, whereas global degree measures connectivity in the whole human protein interaction network. Similarly, we define local and global measures for eigenvector centrality and betweenness.

To use this as a normalization, first, we filter for proteins in the the top five percent of local degree, eigenvector centrality and betweenness. This results in 73 proteins for each metric. Second, to calculate the adjusted centrality metrics we divide the local by the global value. This results in three lists of proteins that are important specifically for HIV regarding these three metrics (see Table 6 and 7).

### Network motif detection

The HIV-Host protein interaction network was analyzed for network motifs using a motif detection algorithm implemented in Prolog (see additional files 13, 14, 15 and 16). The prolog programming language presents a useful alternative for network motif finding as the definition and detection of network patterns is highly intuitive (prolog is a declarative language used for logic programming). In contrast to the motif detection tools MAVisto [61] and Mfinder [36]), our implementation in Prolog and the FANMOD [62] motif finding tool are able to find any annotated network pattern consisting of any number of nodes and edges. This means that we are able to specify the type of edges and nodes, thereby distinguishing between different functional motifs even though they have the same topology (i.e. distinguishing between regulatory and activation/inhibition motifs). Motif detection was carried out for all possible two and three node patterns. To determine the significance of the observed motifs, motif detection was repeated on one thousand randomized networks using a strict randomization algorithm. This to ensure an unchanged connectivity distribution.

#### Randomization Algorithm

Fully randomized networks would make any found network motif to be significant. For this reason a randomized network should be as similar to the original network as possible, yet randomized. In [2,3,36] this is achieved by introducing a rewiring algorithm that iteratively switches the sources or targets of two random edges until the network is sufficiently randomized. This results in a network where the edges are randomized without changing the number of nodes or edges and without changing the degree distribution of the network. In our approach we used a similar algorithm (see Figure 6) for randomizing the networks. Because edges can be of different type, we either switch the sources or targets of two randomly chosen edges with equal probability.

#### Significance

As described in [3,36] the significance of network motifs is determined using the *P*_*value *_and *Z*_*score *_which are calculated using the number of a specific motif found in the original network (*N*_*real*_) and the average number found in the randomized networks (*N*_*rand*_) with standard deviation (*SD*). A network motif is found to be significant if the probability of finding the motif *N*_*real *_times in the randomized networks (*P*_*value*_) is smaller than 0.02 and the number of standard deviations *N*_*real *_is removed from *N*_*rand *_is at least 2. As a result the network motifs that are found to be significant can not just be attributed to randomness.

## Abbreviations

HIV: Human Immunodeficiency Virus; NCBI: The National Center for Biotechnology Information; NIAID: The National Institute of Allergy and Infectious Diseases; GO: Gene Ontology; HDF: HIV Dependency Factors.

## Authors' contributions

DD conceived the project. DD, GE were involved in developing the project. DD performed data processing and computation. GE and DD performed the analyses and wrote the paper. PS is the principal Investigator on the EU grants that funded the project. PS monitored the whole framework. CABB, PS provided constructive discussions and revised the manuscript. All the authors have read and agreed to the manuscript.

## Supplementary Material

Additional file 1 **Protein interaction network of HIV proteins and HDFs**. Intended to be opened in IGraph or any other graph analysis software that supports GML files. Click here for file

Additional file 2 **Protein interaction network of HDFs and non-HDF human proteins**. Intended to be opened in IGraph or any other graph analysis software that supports GML files. Click here for file

Additional file 3 **High resolution picture of the HIV-HDF network generated with Cytoscape, in spring embedded layout**. Click here for file

Additional file 4 **Cytoscape data file of the HIV-HDF networks**. Click here for file

Additional file 5 **HIV-human connectivities for regulatory, activation/inhibition and binds interactions**. A: Number of HIV-human interactions for each HIV protein, B: Normalized relative number of HIV-human interactions for each HIV protein. The y-axis enumerates the n-fold representation of interactions per HIV protein divided by the relative number of interactions of the respective protein in the total network. An n-fold representation of *n *> 1 shows an over-representation, whereas *n *< 1 signifies an under-representation. Click here for file

Additional file 6 **Spreadsheet document with all the proteins from the network centrality analysis**. Click here for file

Additional file 7 **The proteins from proteasome network and their first and second neighbors**. Click here for file

Additional file 8 **High resolution image of the proteasomal network generated with Cytoscape, in spring embedded layout**. Click here for file

Additional file 9 **Heat-map image from proteasomal network to annotate for the common GeneOntology terms associated with cell cycle in cluster-2**. Click here for file

Additional file 10 **Heat-map image from proteasomal network to annotate for the common GeneOntology terms associated with regulation in cluster-2**. Click here for file

Additional file 11 **Heat-map image from proteasomal network to annotate for the common GeneOntology terms in cluster-3**. Click here for file

Additional file 12 **Tables of all found significantly over-represented network motifs in the regulatory and activation/inhibition sub-networks**. Also included are tables of specific protein involvement and Gene Ontology analysis of the motifs. Click here for file

Additional file 13 **Prolog file containing the HIV-HDF regulatory network**. Click here for file

Additional file 14 **Prolog file containing the HIV-HDF activation/inhibition network**. Click here for file

Additional file 15 **Prolog script with an implementation of the motif detection and network randomization algorithm for the regulatory network**. Click here for file

Additional file 16 **Prolog script with an implementation of the motif detection and network randomization algorithm for the activation/inhibition network**. Click here for file
