# Supplementary material for: Identifying potential survival strategies of HIV-1 through virus-host protein interaction networks
Source: BMC Syst Biol. 2010 Jul 15;4:96. doi: 10.1186/1752-0509-4-96 (PMC2913931; doi:10.1186/1752-0509-4-96)
Supplement: Additional file 3 — High resolution picture of the HIV-HDF network generated with Cytoscape, in spring embedded layout. [file 1752-0509-4-96-S3.PDF]

| ID | motif | $N_{real}$ | $N_{rand} \pm SD$ | $P_{value}$ | $Z_{score}$ |
|---|---|---|---|---|---|
| 1 | 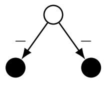 | 1 | $0.01 \pm 0.1$ | 0 | 9.9 |
| 2 | 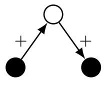 | 15 | $7 \pm 4$ | 0.0134 | 2.21 |
| 3 | 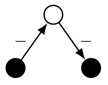 | 8 | $3 \pm 2$ | 0.0183 | 2.09 |
| 4 | 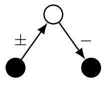 | 2 | $0.3 \pm 0.8$ | 0.0167 | 2.13 |
| 5 | 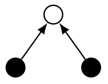 | 577 | $425 \pm 20$ | $3.1 \cdot 10^{-14}$ | 7.50 |
| 6 | 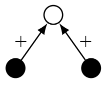 | 200 | $163 \pm 13$ | $2.5 \cdot 10^{-3}$ | 2.80 |
| 7 | 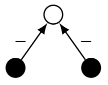 | 115 | $51 \pm 10$ | $1.6 \cdot 10^{-10}$ | 6.29 |
| 8 | 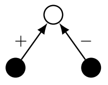 | 336 | $278 \pm 25$ | $9.4 \cdot 10^{-3}$ | 2.35 |
| 9 | 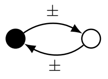 | 4 | $0.7 \pm 0.8$ | $1.7 \cdot 10^{-5}$ | 4.14 |
| 10 | 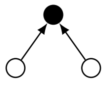 | 2341 | $1569 \pm 156$ | $3.9 \cdot 10^{-7}$ | 4.94 |
| 11 | 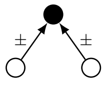 | 2155 | $1246 \pm 128$ | $6.2 \cdot 10^{-13}$ | 7.10 |
| 12 | 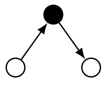 | 24466 | $21914 \pm 927$ | $2.9 \cdot 10^{-3}$ | 2.8 |

Table 1: Significantly over-represented network motifs in HIV-host regulatory network. Black nodes are HIV proteins and white nodes are human proteins. Interactions can either be activations/up-regulations (+), inhibitions/down-regulations (−), signaling/regulation (±), or both (arrow without sign). $N_{real}$ is the number of specific motifs found. $N_{rand} \pm SD$ is the average number and standard deviation of the motif found in one thousand randomized networks. $P_{value}$ is the probability that $N_{real}$ or more motifs are found in the randomized networks. $Z_{score}$ is the number of standard deviations $N_{rand}$ differs from $N_{real}$. Network motifs were classified as significant when $P_{value} < 0.02$ and $Z_{score} > 2$.

| ID | motif | $N_{real}$ | $N_{rand} \pm SD$ | $P_{value}$ | $Z_{score}$ |
|---|---|---|---|---|---|
| 13 | 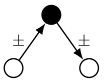 | 482 | $374 \pm 48$ | 0.0123 | 2.25 |
| 14 | 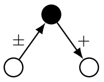 | 14718 | $12457 \pm 572$ | $3.9 \cdot 10^{-5}$ | 3.95 |
| 15 | 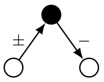 | 8348 | $6709 \pm 377$ | $6.8 \cdot 10^{-6}$ | 4.35 |
| 16 | 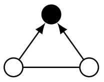 | 74 | $16 \pm 6$ | 0 | 9.97 |
| 17 | 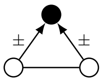 | 71 | $12 \pm 5$ | 0 | 11.0 |
| 18 | 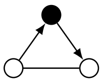 | 322 | $218 \pm 35$ | $1.6 \cdot 10^{-3}$ | 2.94 |
| 19 | 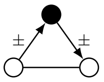 | 22 | $4 \pm 2$ | $1.11 \cdot 10^{-16}$ | 8.28 |
| 20 | 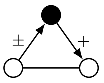 | 181 | $124 \pm 23$ | $7.7 \cdot 10^{-3}$ | 2.43 |
| 21 | 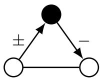 | 107 | $67 \pm 15$ | $2.8 \cdot 10^{-3}$ | 2.77 |
| 22 | 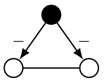 | 130 | $90 \pm 19$ | 0.0175 | 2.11 |
| 23 | 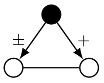 | 30 | $16 \pm 6$ | 0.0135 | 2.21 |
| 24 | 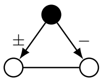 | 20 | $10 \pm 5$ | 0.0147 | 2.18 |

Table 2: Significantly over-represented network motifs in HIV-host regulatory network. Black nodes are HIV proteins and white nodes are human proteins. Interactions can either be activations/up-regulations (+), inhibitions/down-regulations (−), signaling/regulation (±), or both (arrow without sign). $N_{real}$ is the number of specific motifs found. $N_{rand} \pm SD$ is the average number and standard deviation of the motif found in one thousand randomized networks. $P_{value}$ is the probability that $N_{real}$ or more motifs are found in the randomized networks. $Z_{score}$ is the number of standard deviations $N_{rand}$ differs from $N_{real}$. Network motifs were classified as significant when $P_{value} < 0.02$ and $Z_{score} > 2$.

| ID | motif | $N_{real}$ | $N_{rand} \pm SD$ | $P_{value}$ | $Z_{score}$ |
|---|---|---|---|---|---|
| 25 | 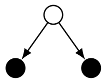 | 35 | $23 \pm 5$ | 0.0114 | 2.28 |
| 26 | 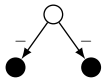 | 34 | $21 \pm 6$ | 0.0153 | 2.16 |
| 27 | 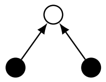 | 304 | $240 \pm 15$ | $6.9 \cdot 10^{-6}$ | 4.35 |
| 28 | 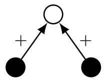 | 167 | $78 \pm 11$ | $1.1 \cdot 10^{-16}$ | 8.19 |
| 29 | 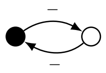 | 22 | $15 \pm 3$ | 0.0140 | 2.20 |
| 30 | 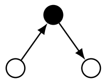 | 42912 | $40680 \pm 1031$ | 0.0151 | 2.17 |
| 31 | 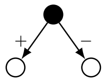 | 20912 | $19820 \pm 511$ | 0.0166 | 2.13 |
| 32 | 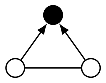 | 77 | $39 \pm 11$ | $1.9 \cdot 10^{-4}$ | 3.56 |
| 33 | 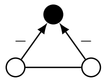 | 72 | $36 \pm 10$ | $1.7 \cdot 10^{-4}$ | 3.59 |
| 34 | 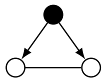 | 659 | $402 \pm 44$ | $2.7 \cdot 10^{-9}$ | 5.84 |
| 35 | 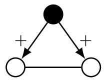 | 324 | $131 \pm 27$ | $4.5 \cdot 10^{-13}$ | 7.14 |
| 36 | 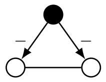 | 175 | $74 \pm 17$ | $1.4 \cdot 10^{-9}$ | 5.95 |

Table 3: Significantly over-represented network motifs in HIV-host signaling network. Black nodes are HIV proteins and white nodes are human proteins. Interactions can either be activations/up-regulations (+), inhibitions/down-regulations (−), signaling/regulation (±), or both (arrow without sign). $N_{real}$ is the number of specific motifs found. $N_{rand} \pm SD$ is the average number and standard deviation of the motif found in one thousand randomized networks. $P_{value}$ is the probability that $N_{real}$ or more motifs are found in the randomized networks. $Z_{score}$ is the number of standard deviations $N_{rand}$ differs from $N_{real}$. Network motifs were classified as significant when $P_{value} < 0.02$ and $Z_{score} > 2$.

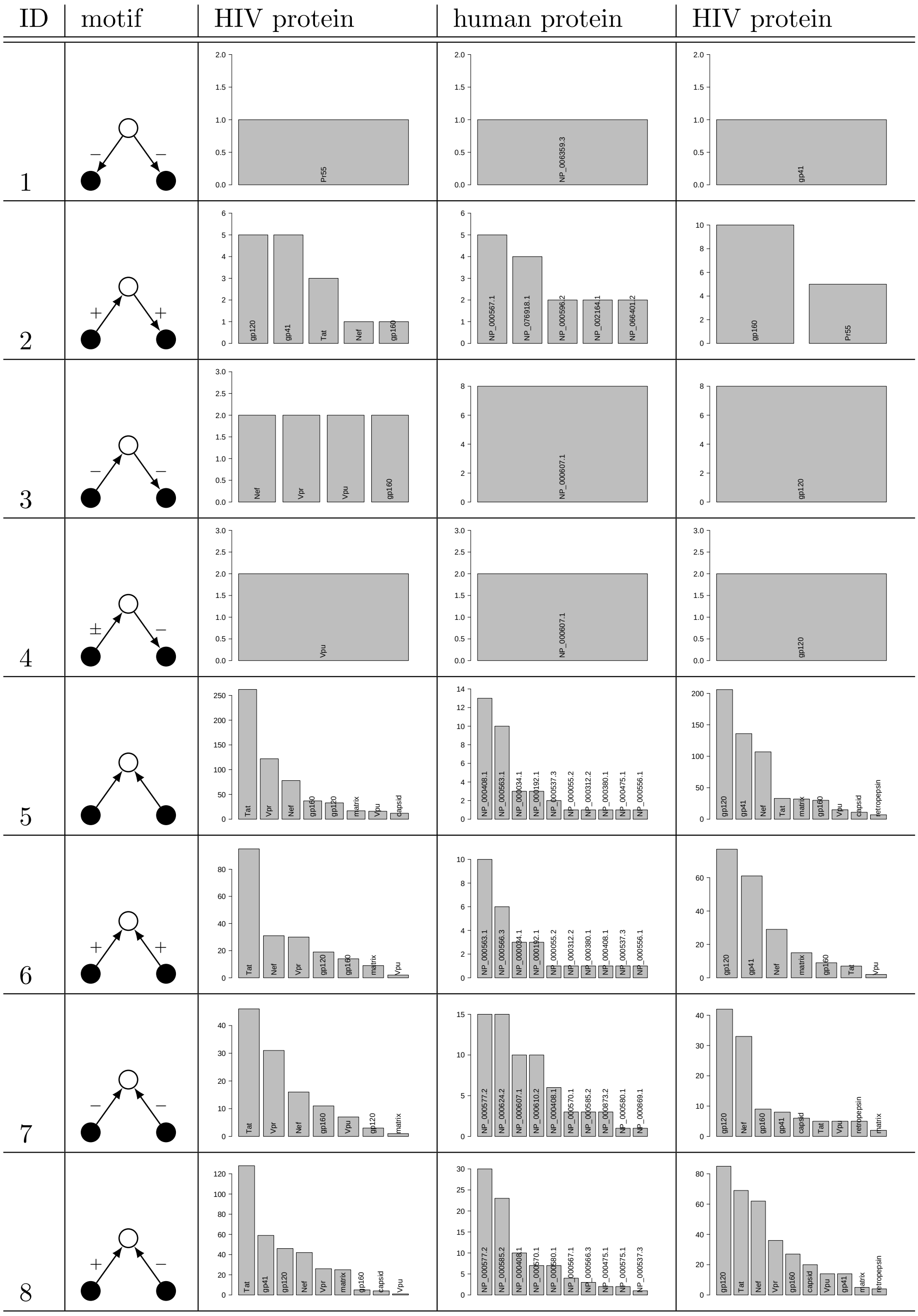


Table 4: Involvement of HIV and human proteins in network motifs of HIV-host regulatory network. HIV nodes in symmetric motifs share the same distribution. Of the human proteins at most only the 10 most frequent ones are shown. Human proteins are identified with a RefSeq protein accession number.

| ID | motif | HIV protein | human protein | HIV protein |
|---|---|---|---|---|
| 9 | 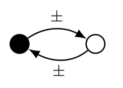 | 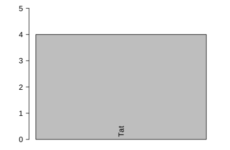 | 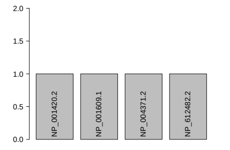 | |
| 10 | 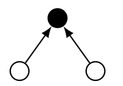 | 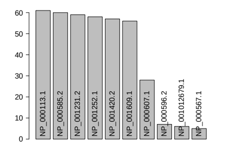 | 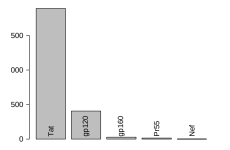 | 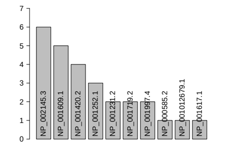 |
| 11 | 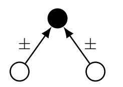 | 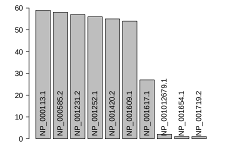 | 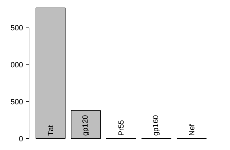 | 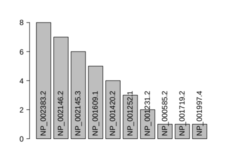 |
| 12 | 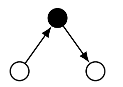 | 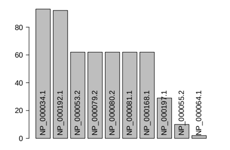 | 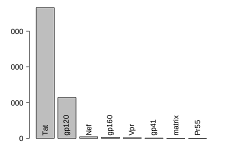 | 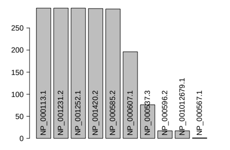 |
| 13 | 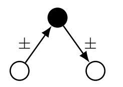 | 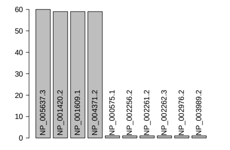 | 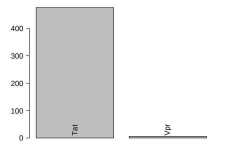 | 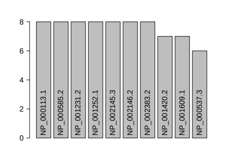 |
| 14 | 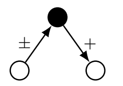 | 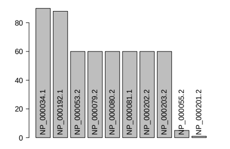 | 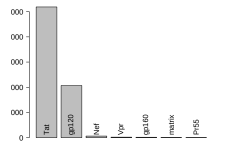 | 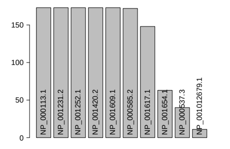 |
| 15 | 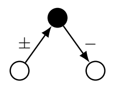 | 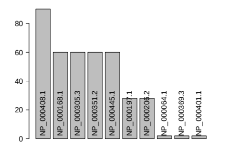 | 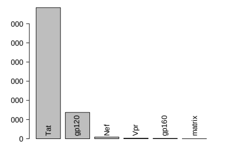 | 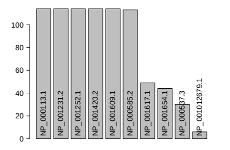 |
| 16 | 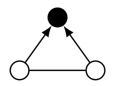 | 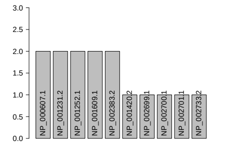 | 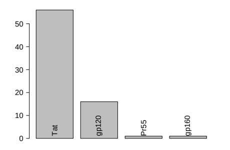 | 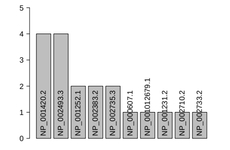 |

Table 5: Involvement of HIV and human proteins in network motifs of HIV-host regulatory network. HIV nodes in symmetric motifs share the same distribution. Of the human proteins at most only the 10 most frequent ones are shown. Human proteins are identified with a RefSeq protein accession number.

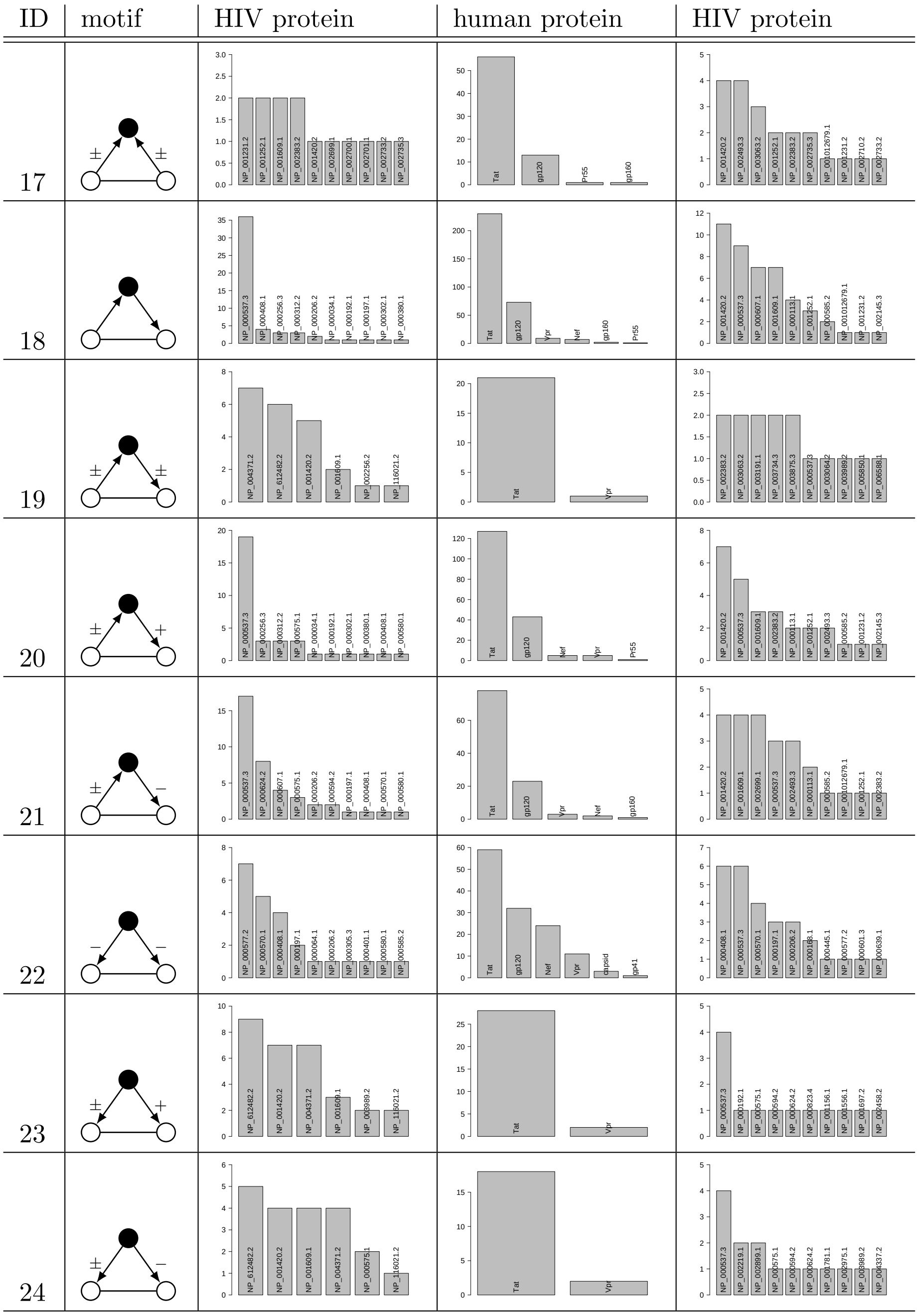


Table 6: Involvement of HIV and human proteins in network motifs of HIV-host regulatory network. HIV nodes in symmetric motifs share the same distribution. Of the human proteins at most only the 10 most frequent ones are shown. Human proteins are identified with a RefSeq protein accession number.

| ID | motif | HIV protein | human protein | HIV protein |
|---|---|---|---|---|
| 25 | 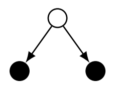 | 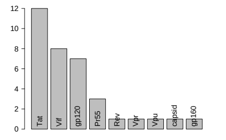 | 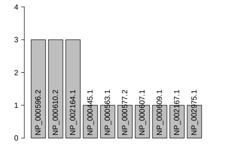 | 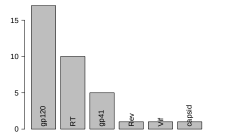 |
| 26 | 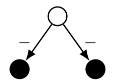 | 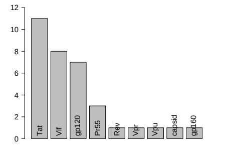 | 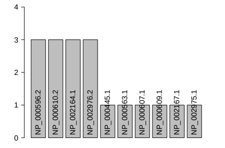 | 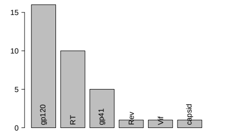 |
| 27 | 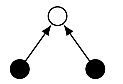 | 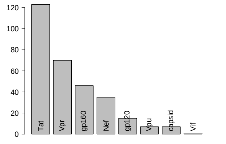 | 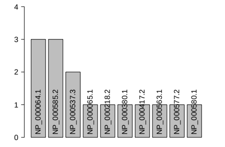 | 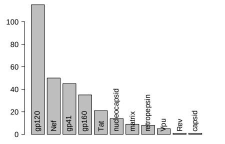 |
| 28 | 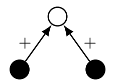 | 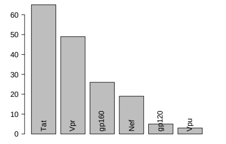 | 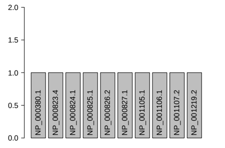 | 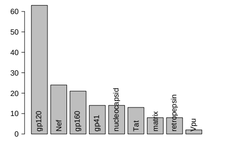 |
| 29 | 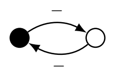 | 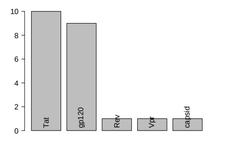 | 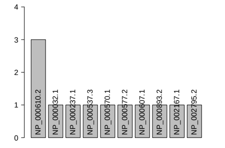 | |
| 30 | 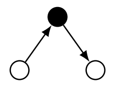 | 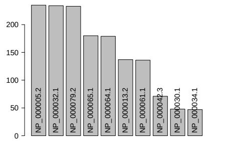 | 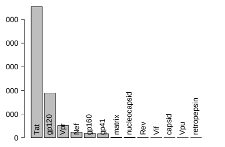 | 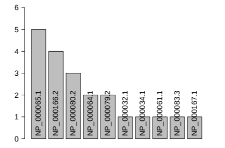 |

Table 7: Involvement of HIV and human proteins in network motifs of HIV-host signalling network. HIV nodes in symmetric motifs share the same distribution. Of the human proteins at most only the 10 most frequent ones are shown. Human proteins are identified with a RefSeq protein accession number.

<table>
<tr><th>ID</th><th>motif</th><th>HIV protein</th><th>human protein</th><th>HIV protein</th></tr>
<tr><td>31</td><td>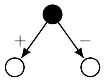
</td><td>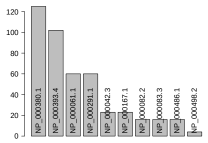
</td><td>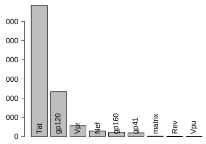
</td><td>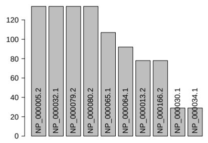
</td></tr>
<tr><td>32</td><td>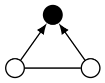</td><td>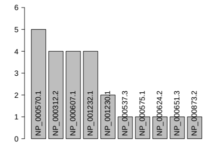
</td><td>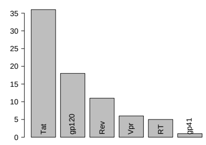
</td><td>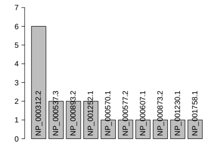
</td></tr>
<tr><td>33</td><td>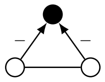
</td><td>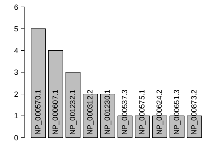
</td><td>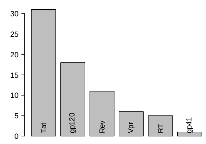
</td><td>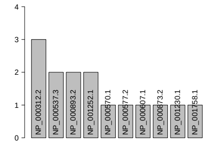
</td></tr>
<tr><td>34</td><td>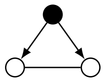</td><td>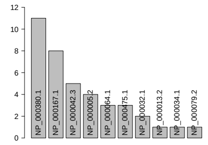
</td><td>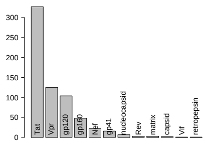
</td><td>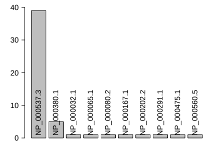
</td></tr>
<tr><td>35</td><td>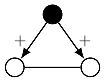
</td><td>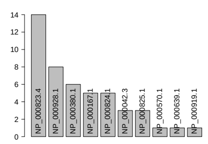
</td><td>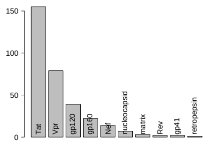
</td><td>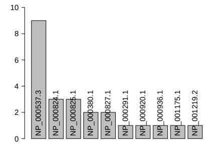
</td></tr>
<tr><td>36</td><td>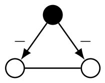
</td><td>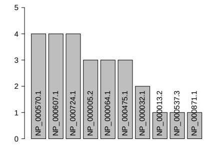
</td><td>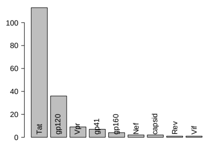
</td><td>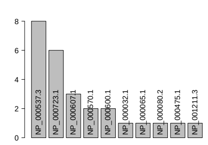
</td></tr>
</table>

Table 8: Involvement of HIV and human proteins in network motifs of HIV-host signalling network. HIV nodes in symmetric motifs share the same distribution. Of the human proteins at most only the 10 most frequent ones are shown. Human proteins are identified with a RefSeq protein accession number.

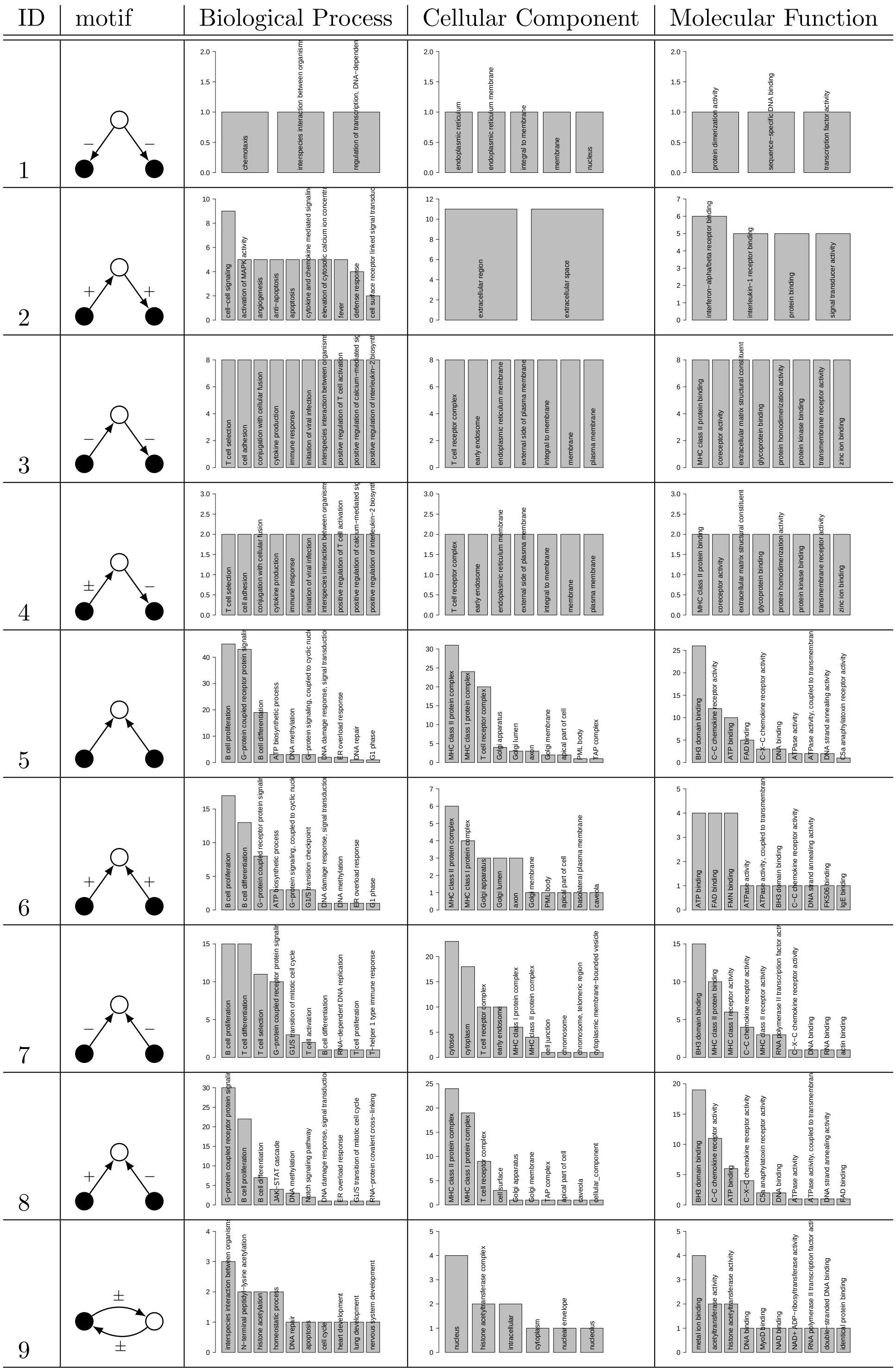


Table 9: Gene Ontology annotation of human proteins involved in network motifs of HIV-human regulatory network.

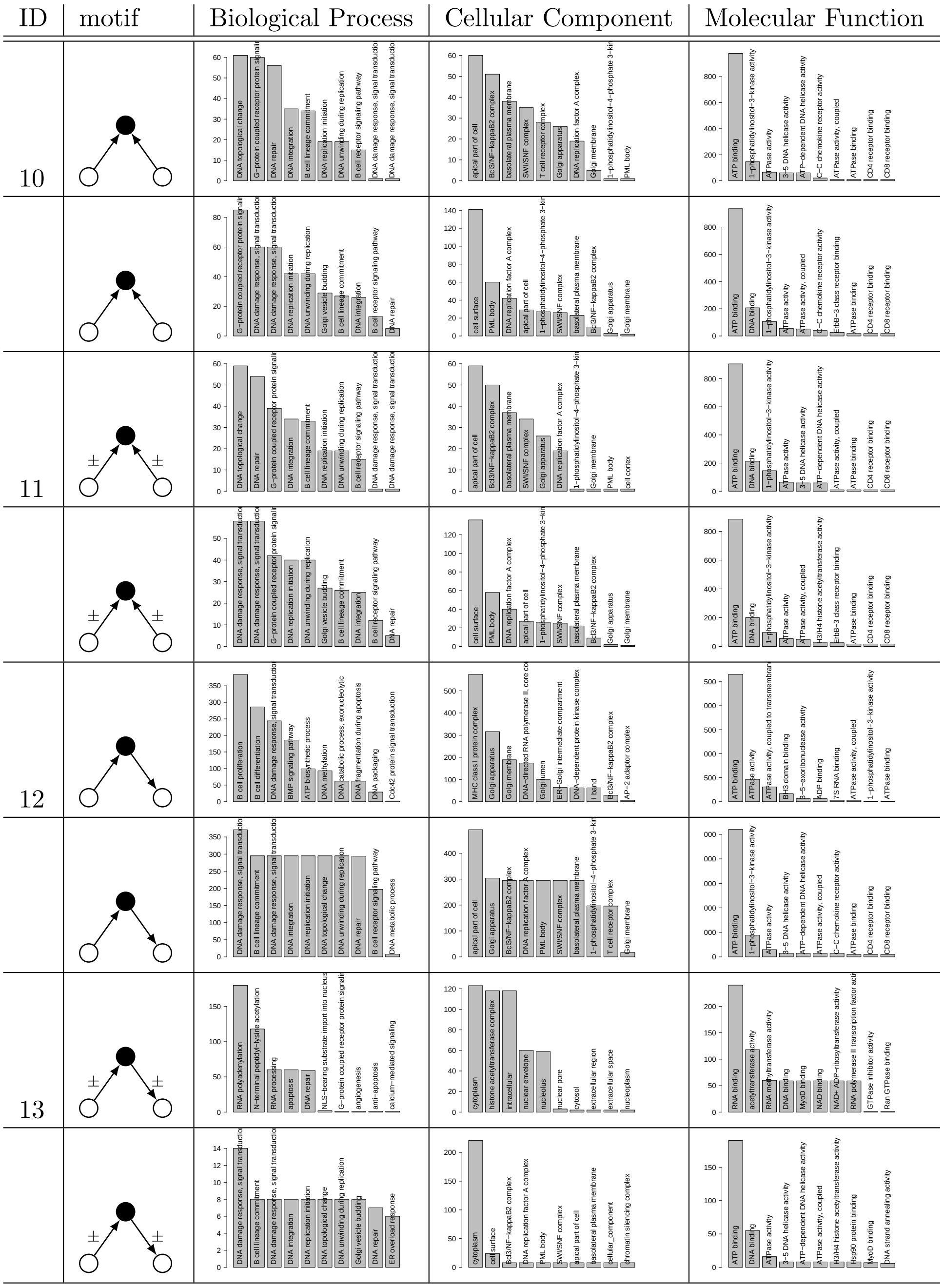


Table 10: Gene Ontology annotation of human proteins involved in network motifs of HIV-human regulatory network.

| ID | motif | Biological Process | Cellular Component | Molecular Function |
|---|---|---|---|---|
| 14 | 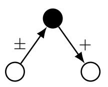 | 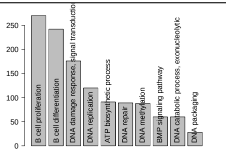 | 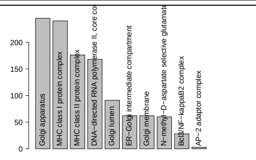 | 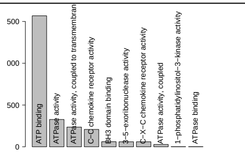 |
| | 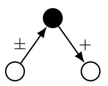 | 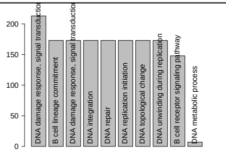 | 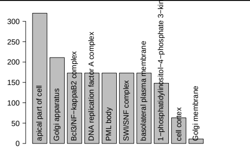 | 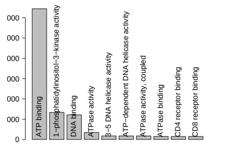 |
| 15 | 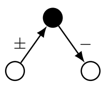 | 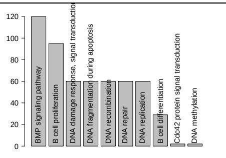 | 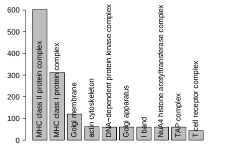 | 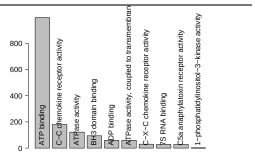 |
| | 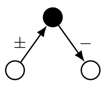 | 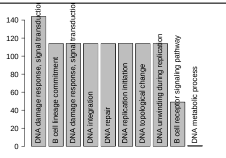 | 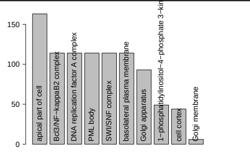 | 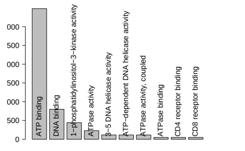 |
| 16 | 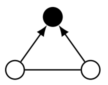 | 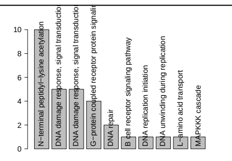 | 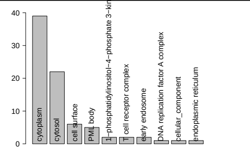 | 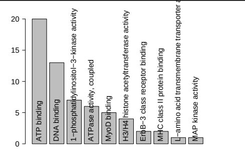 |
| | 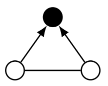 | 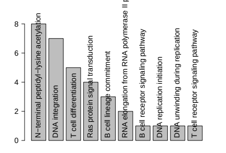 | 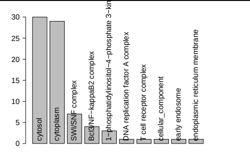 | 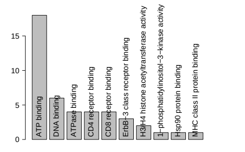 |
| 17 | 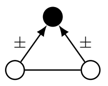 | 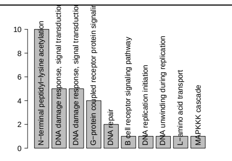 | 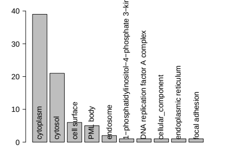 | 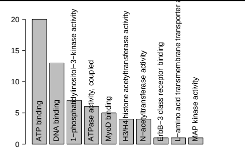 |
| | 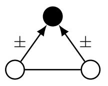 | 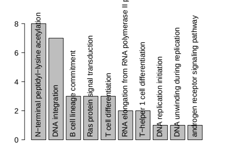 | 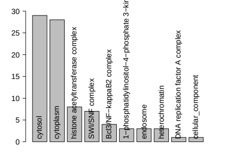 | 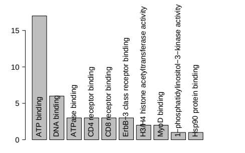 |

Table 11: Gene Ontology annotation of human proteins involved in network motifs of HIV-human regulatory network.

| ID | motif | Biological Process | Cellular Component | Molecular Function |
|---|---|---|---|---|
| 18 | 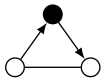 | 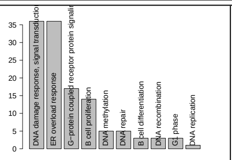 | 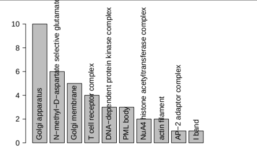 | 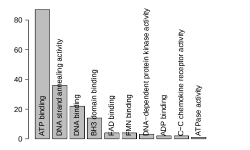 |
| | 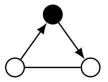 | 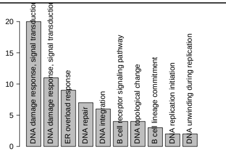 | 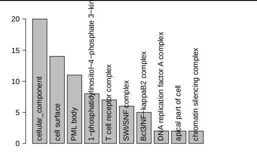 | 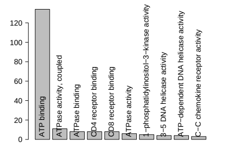 |
| 19 | 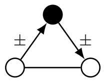 | 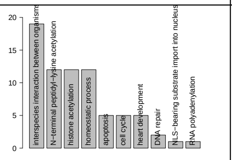 | 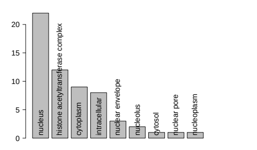 | 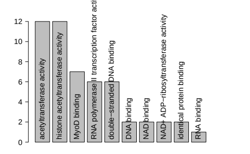 |
| | 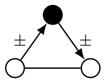 | 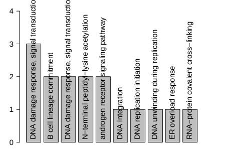 | 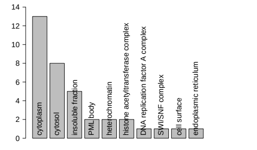 | 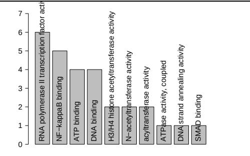 |
| 20 | 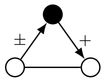 | 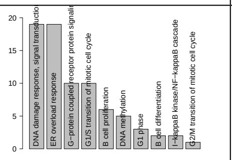 | 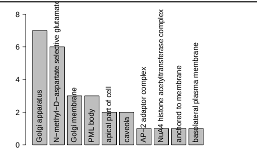 | 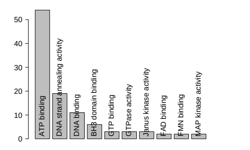 |
| | 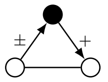 | 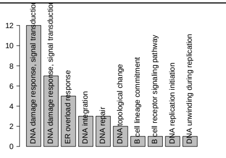 | 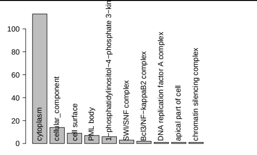 | 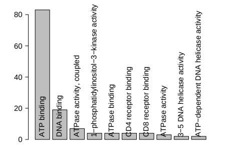 |
| 21 | 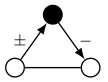 | 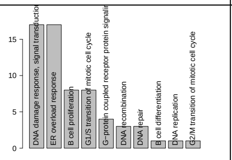 | 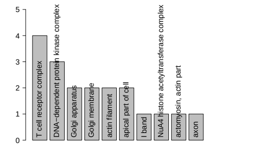 | 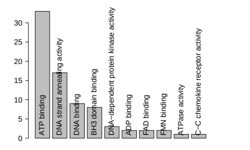 |
| | 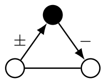 | 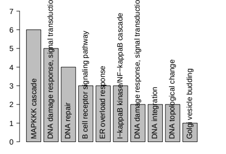 | 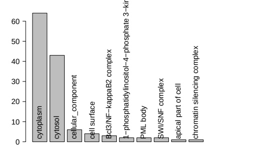 | 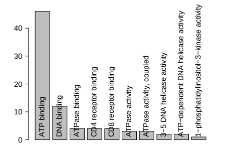 |

Table 12: Gene Ontology annotation of human proteins involved in network motifs of HIV-human regulatory network.

| ID | motif | Biological Process | Cellular Component | Molecular Function |
|---|---|---|---|---|
| 22 | 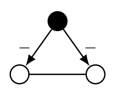 | 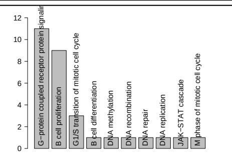 | 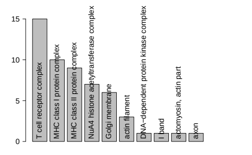 | 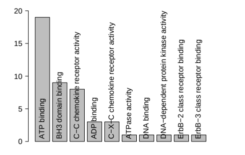 |
| | 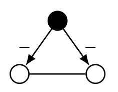 | 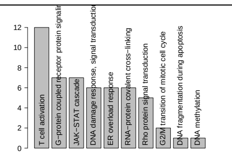 | 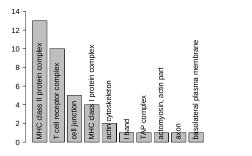 | 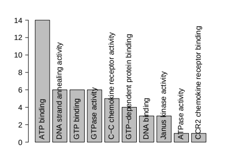 |
| 23 | 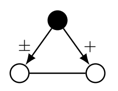 | 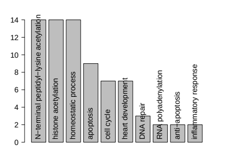 | 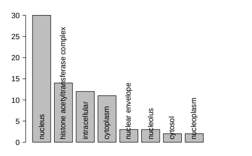 | 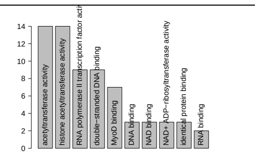 |
| | 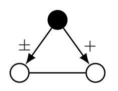 | 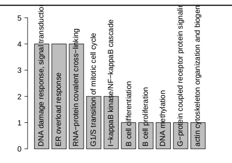 | 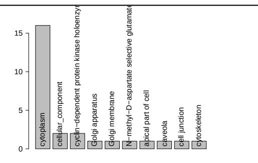 | 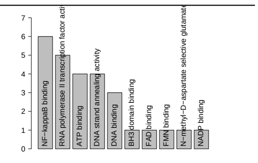 |
| 24 | 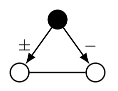 | 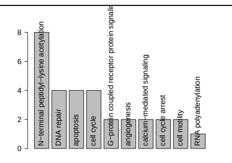 | 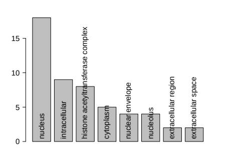 | 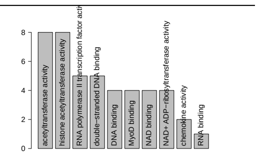 |
| | 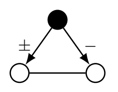 | 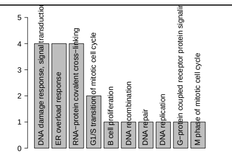 | 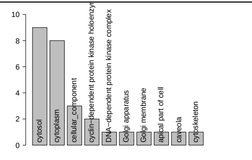 | 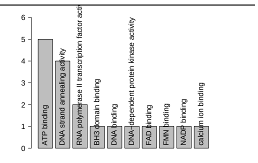 |

Table 13: Gene Ontology annotation of human proteins involved in network motifs of HIV-human regulatory network.

| ID | motif | Biological Process | Cellular Component | Molecular Function |
|---|---|---|---|---|
| 25 | 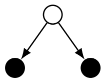 | 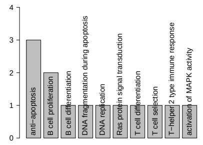 | 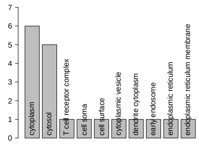 | 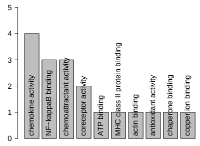 |
| 26 | 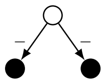 | 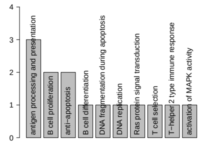 | 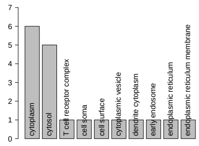 | 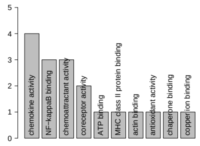 |
| 27 | 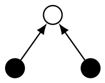 | 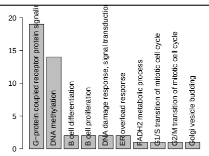 | 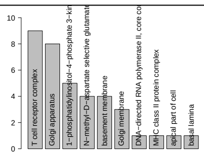 | 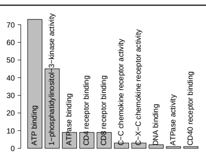 |
| 28 | 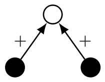 | 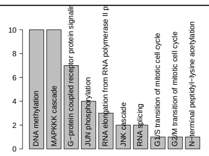 | 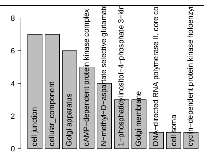 | 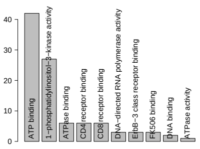 |
| 29 | 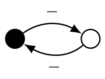 | 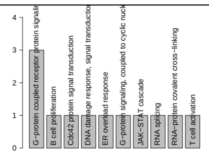 | 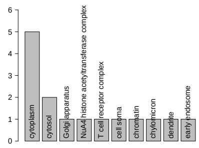 | 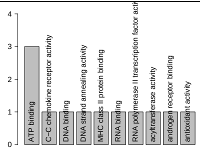 |

Table 14: Gene Ontology annotation of human proteins involved in network motifs of HIV-human signaling network.

| ID | motif | Biological Process | Cellular Component | Molecular Function |
|---|---|---|---|---|
| 30 | 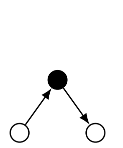 | 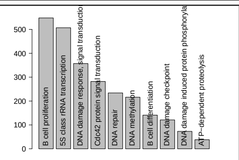 | 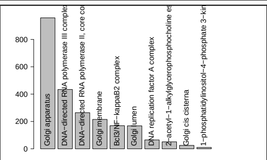 | 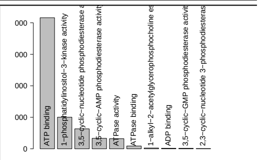 |
| | 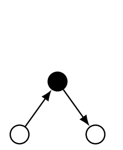 | 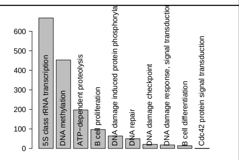 | 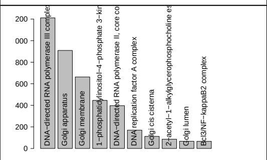 | 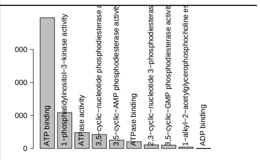 |
| 31 | 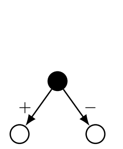 | 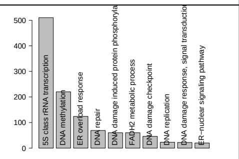 | 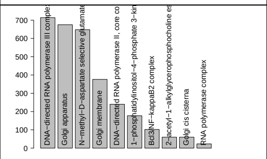 | 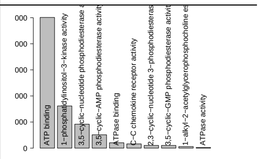 |
| | 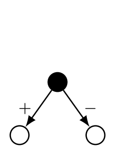 | 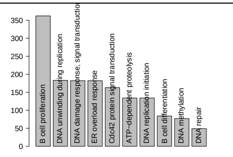 | 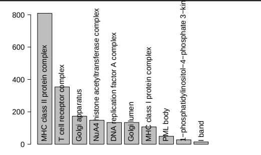 | 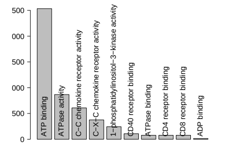 |
| 32 | 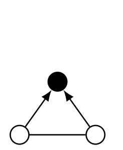 | 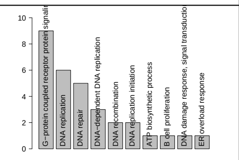 | 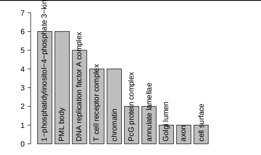 | 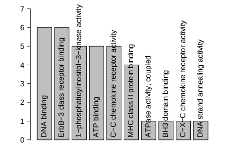 |
| | 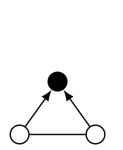 | 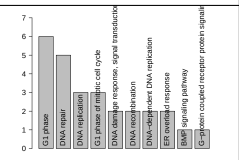 | 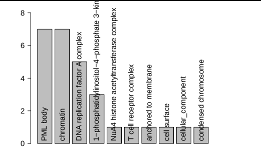 | 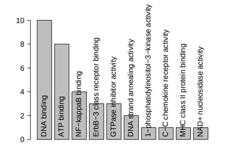 |
| 33 | 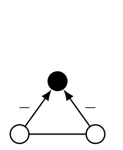 | 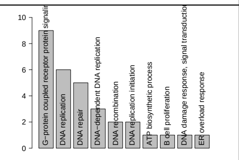 | 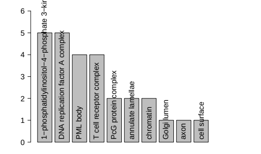 | 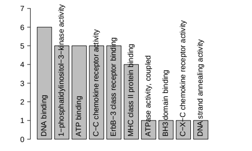 |
| | 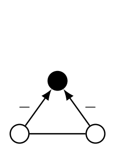 | 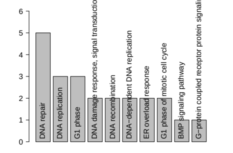 | 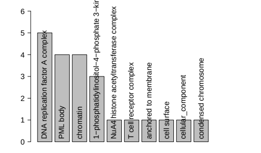 | 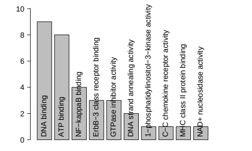 |

Table 15: Gene Ontology annotation of human proteins involved in network motifs of HIV-human signaling network.

| ID | motif | Biological Process | Cellular Component | Molecular Function |
|---|---|---|---|---|
| 34 | 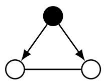 | 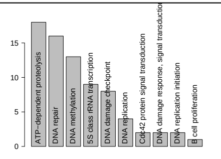 | 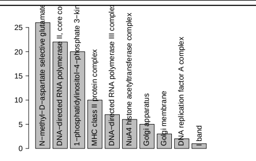 | 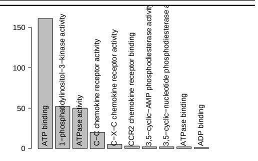 |
| | 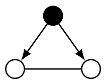 | 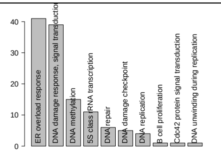 | 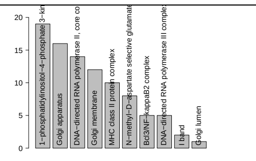 | 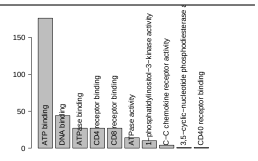 |
| 35 | 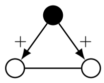 | 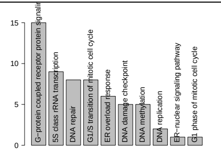 | 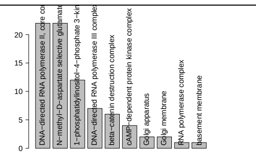 | 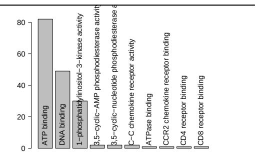 |
| | 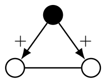 | 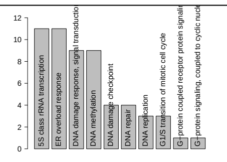 | 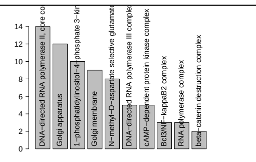 | 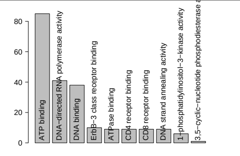 |
| 36 | 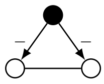 | 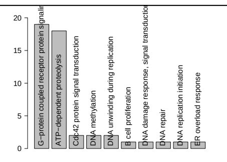 | 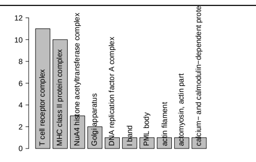 | 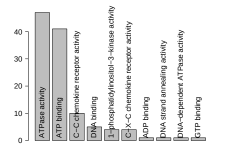 |
| | 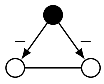 | 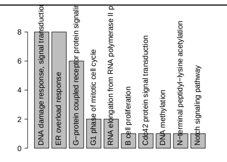 | 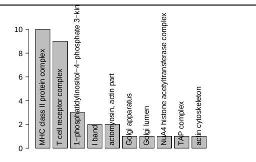 | 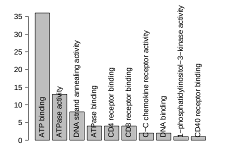 |

Table 16: Gene Ontology annotation of human proteins involved in network motifs of HIV-human signaling network.
