# Supplementary figures and images for: Identifying potential survival strategies of HIV-1 through virus-host protein interaction networks
Source: BMC Syst Biol. 2010 Jul 15;4:96. doi: 10.1186/1752-0509-4-96 (PMC2913931; doi:10.1186/1752-0509-4-96)

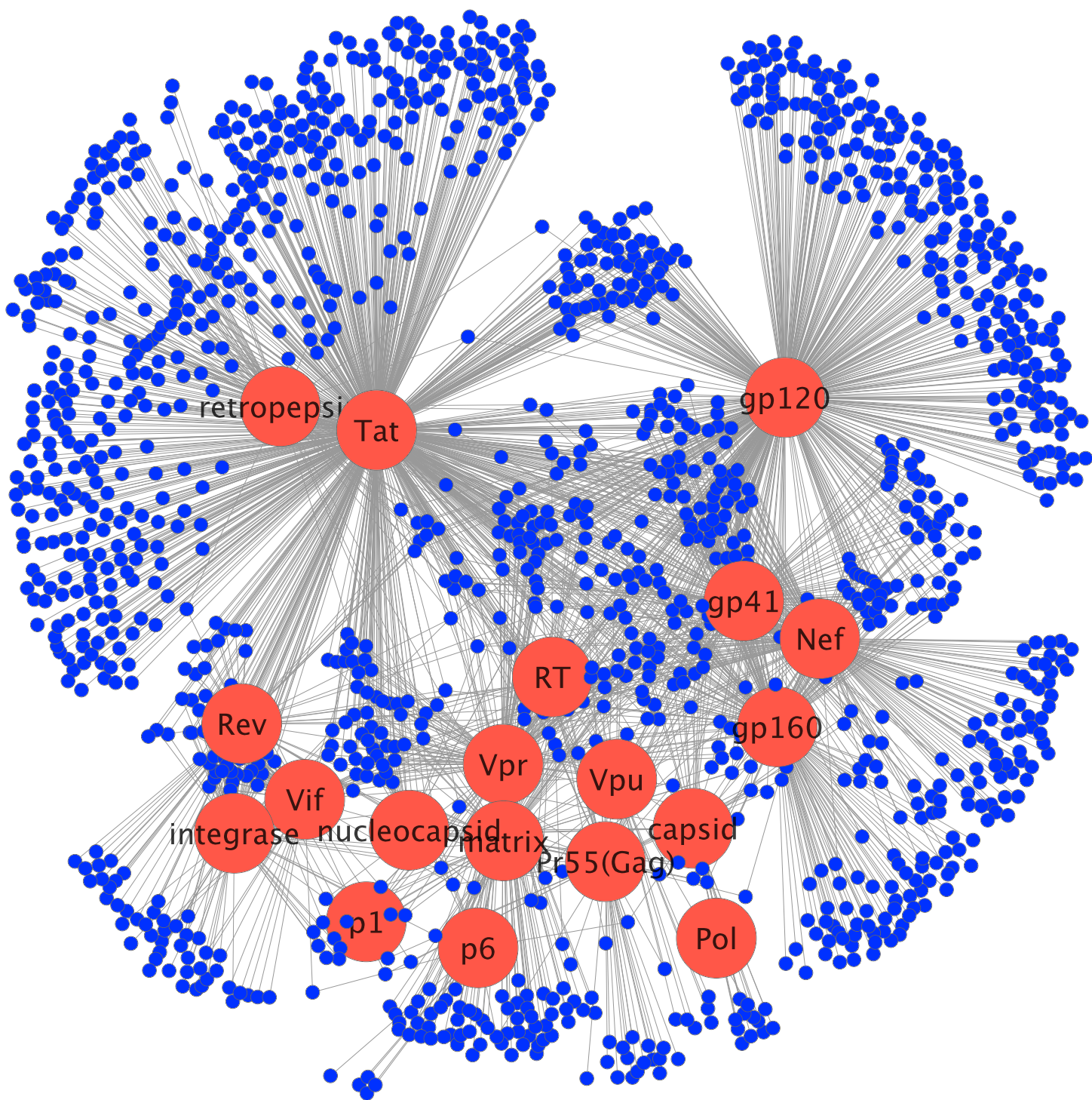

Supplement: Additional file 4 — Cytoscape data file of the HIV-HDF networks. [file 1752-0509-4-96-S4.PDF]

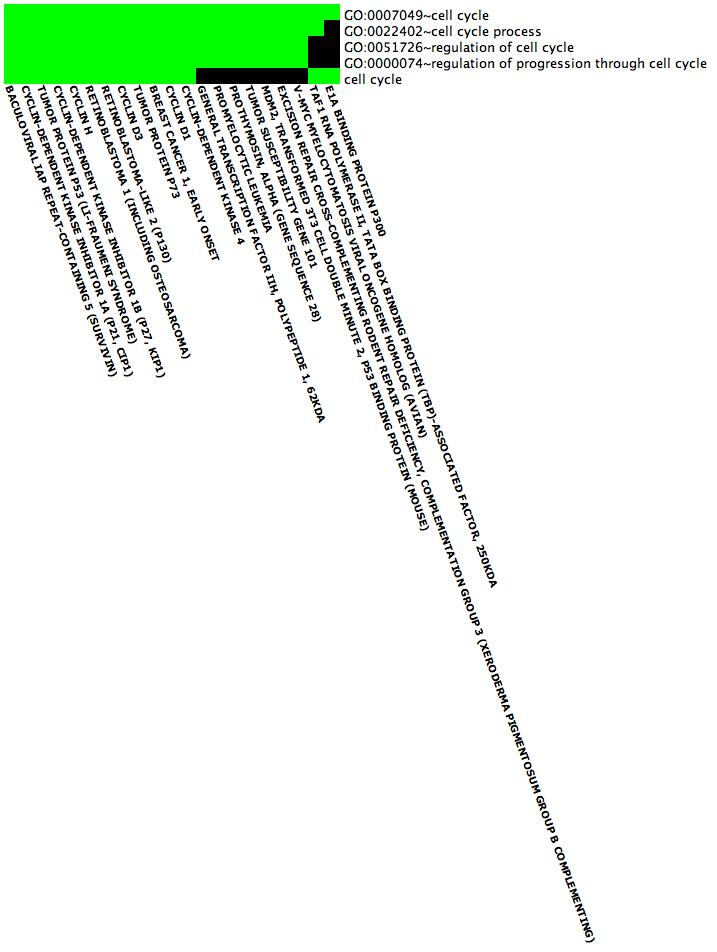

Supplement: Additional file 9 — Heat-map image from proteasomal network to annotate for the common GeneOntology terms associated with cell cycle in cluster-2. [file 1752-0509-4-96-S9.TIFF]

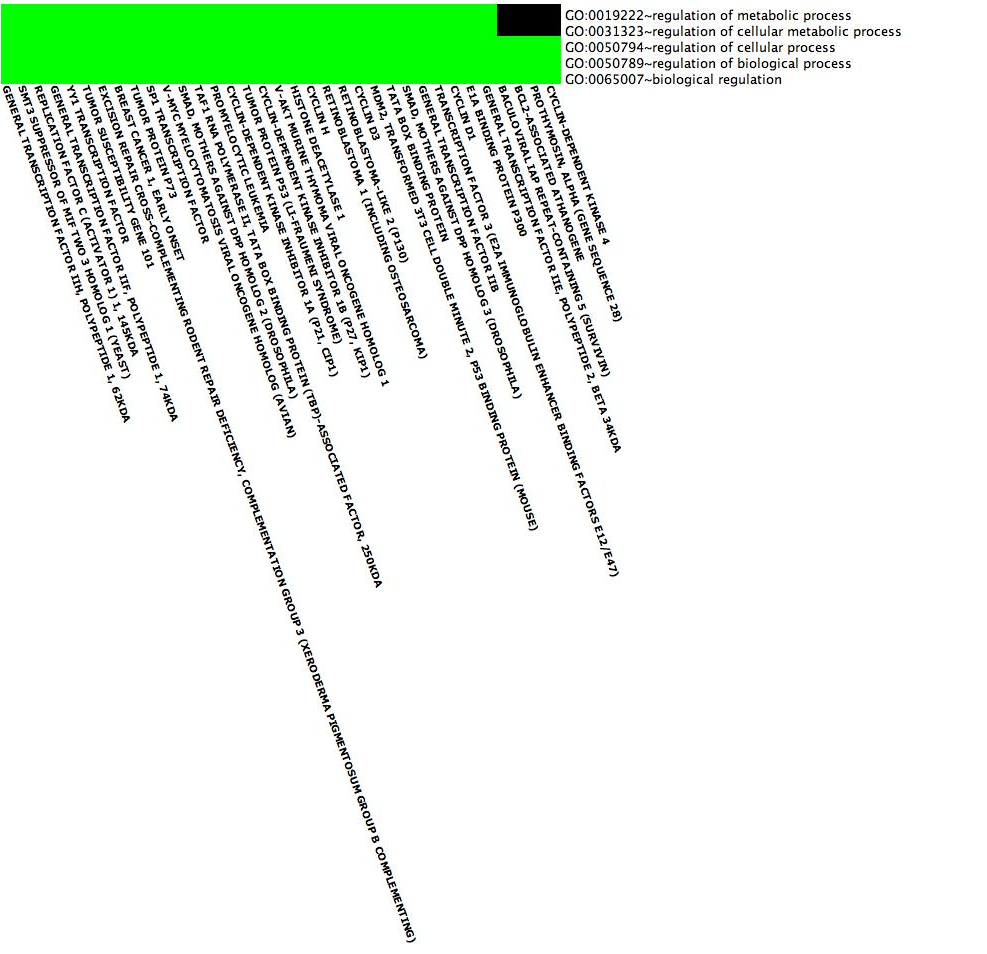

Supplement: Additional file 10 — Heat-map image from proteasomal network to annotate for the common GeneOntology terms associated with regulation in cluster-2. [file 1752-0509-4-96-S10.TIFF]

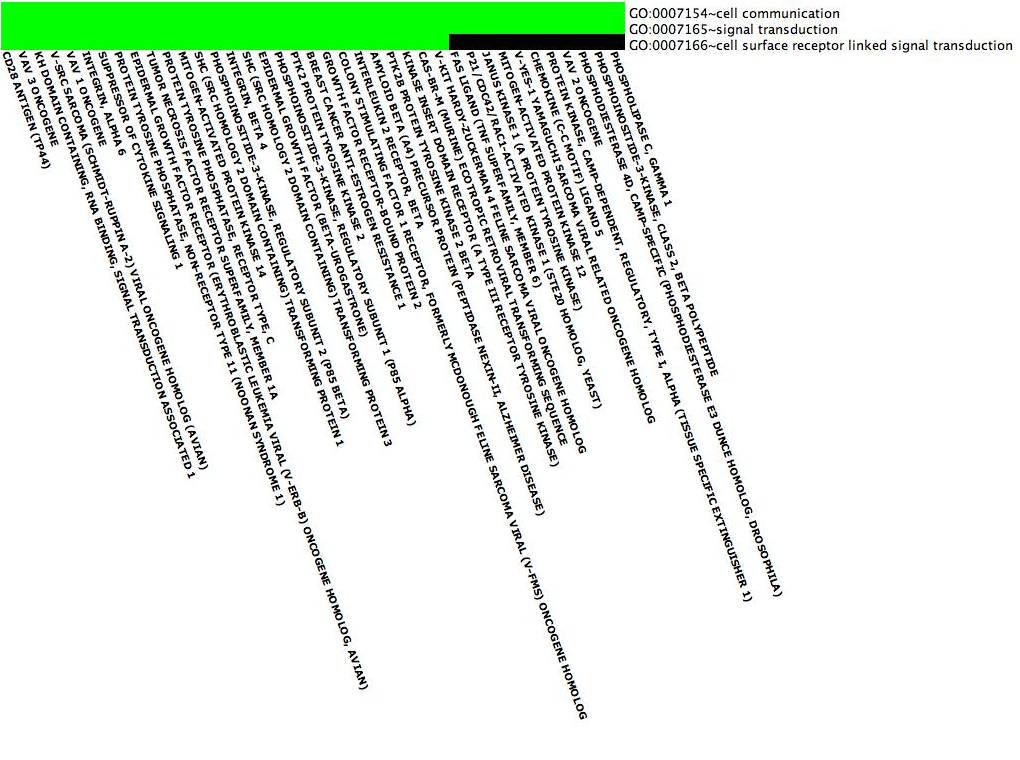

Supplement: Additional file 11 — Heat-map image from proteasomal network to annotate for the common GeneOntology terms in cluster-3. [file 1752-0509-4-96-S11.TIFF]

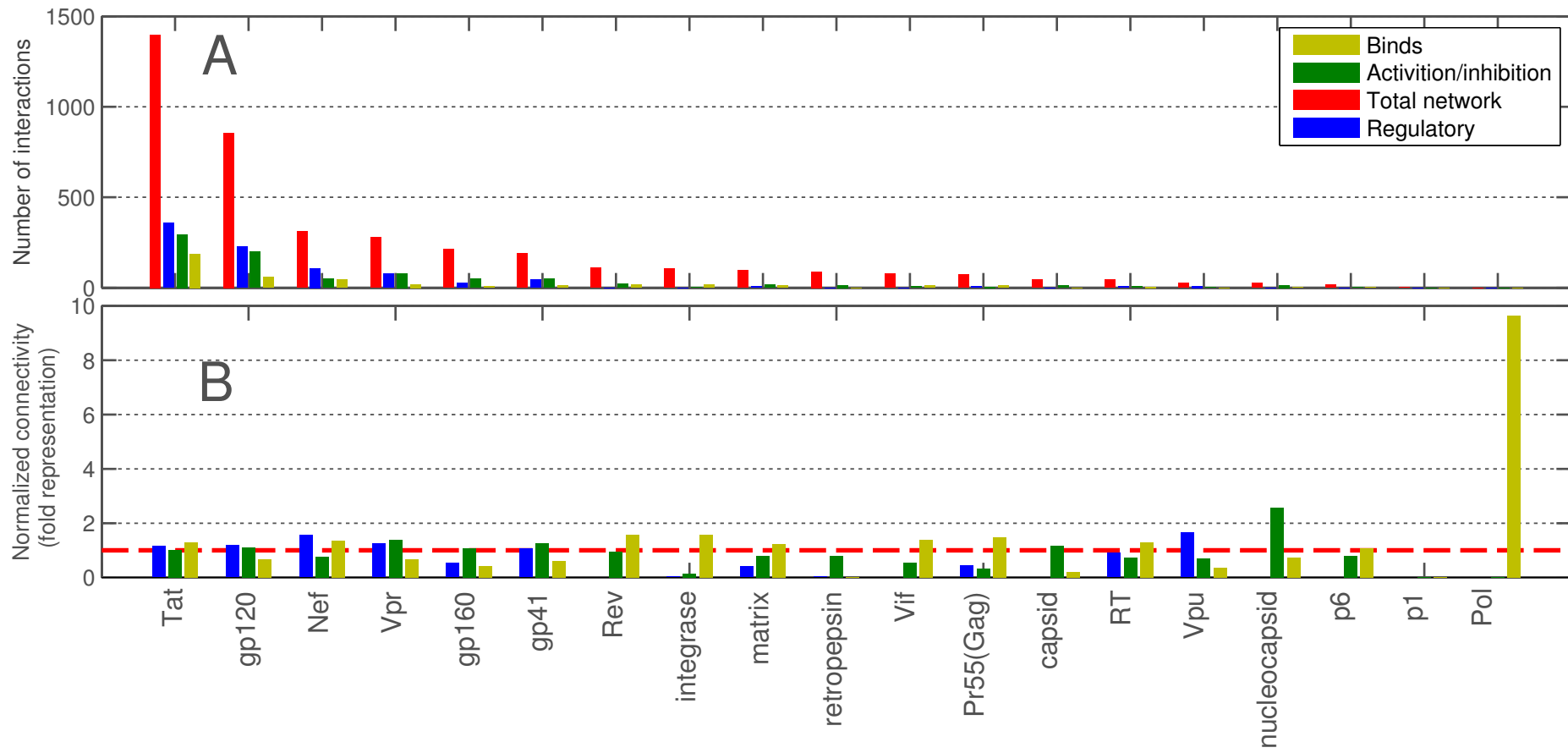

Supplement: Additional file 12 — Tables of all found significantly over-represented network motifs in the regulatory and activation/inhibition sub-networks. Also included are tables of specific protein involvement and Gene Ontology analysis of the motifs. [file 1752-0509-4-96-S12.PDF]
