# Supplementary material for: Identifying potential survival strategies of HIV-1 through virus-host protein interaction networks
Source: BMC Syst Biol. 2010 Jul 15;4:96. doi: 10.1186/1752-0509-4-96 (PMC2913931; doi:10.1186/1752-0509-4-96)
Supplement: Additional file 8 — High resolution image of the proteasomal network generated with Cytoscape, in spring embedded layout. [file 1752-0509-4-96-S8.PDF]

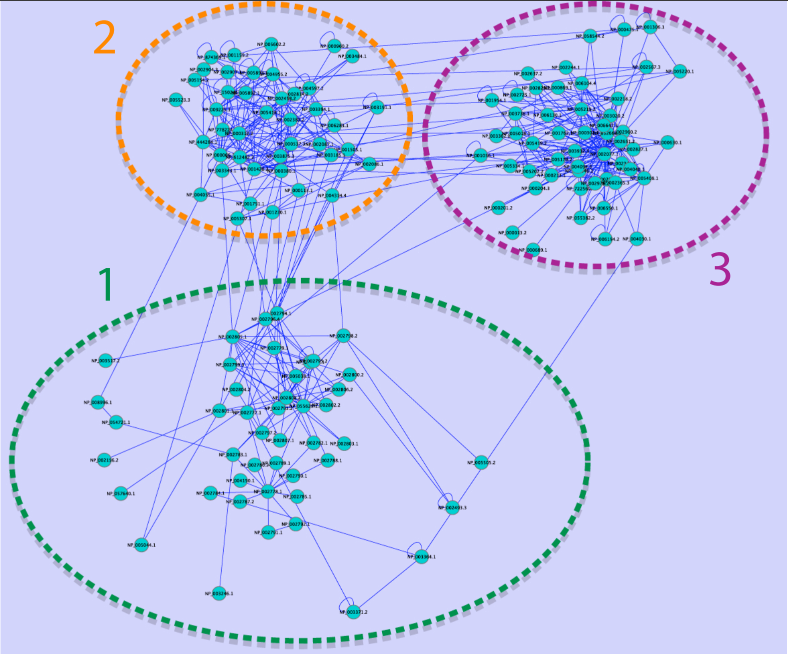

The visualization of the proteasomal network with their first and second neighbors. Resulting network consists of three distinct clusters. 1) The first cluster consists of only proteasomal proteins. 2) Second cluster is associated with regulation of metabolic process (80 percent of all proteins), and regulation of
progression through cell cycle (49 percent of all proteins). The members of this cluster are proteins from highly connected oncogenes Tp53, Tp73, Brca-1 and Rb1. 3) The third cluster is associated with signal transduction and cell communication (both 78 percent).
